# Hypercoordinate iodine for catalytic asymmetric diamination of styrene: insights into the mechanism, role of solvent, and stereoinduction†
†Electronic supplementary information (ESI) available: Cartesian coordinates of all stationary points and other relevant information is provided. See DOI: 10.1039/c9sc01513b


**DOI:** 10.1039/c9sc01513b

**Published:** 2019-06-10

**Authors:** A. Sreenithya, Christopher M. Hadad, Raghavan B. Sunoj

**Affiliations:** a Department of Chemistry , Indian Institute of Technology Bombay , Powai , Mumbai 400076 , India . Email: sunoj@chem.iitb.ac.in; b Department of Chemistry and Biochemistry , The Ohio State University , 100 West 18th Avenue , Columbus , Ohio 43210 , USA

## Abstract

Hypercoordinate iodine has evolved as an impressive class of catalysts for various organic transformations. Extension of this idea to asymmetric applications, such as in the asymmetric difunctionalization of styrene or its derivatives, constitutes an important reaction. In this study, the mechanism and origin of stereoinduction in styrene diamination, with a sulfonimide (HNMs_2_) as the diaminating agent and iodoresorcinol (((iPr)_2_N(CO)-CH(Me)-O)_2_Ar–I) based chiral hypercoordinate iodine as the catalyst, are investigated using density functional theory calculations. The energetically preferred catalytic pathway has been found to involve, among other steps, two very important mechanistic events: (a) the formation of a catalyst–substrate complex by the action of styrene on the catalyst ArI(NMs_2_)_2_, resulting in the displacement of one of the imidates (NMs_2_^–^); and (b) a rebound of the departed imidate on the iodine-bound styrene to form an iodonium ion intermediate with a N–C bond. Explicit interaction of the imidate ion with hexafluoroisopropanol (HFIP), used as a solvent additive, lowers the barrier for the formation of the iodonium ion. The *P* helical fold of the chiral arms of the iodoresorcinol catalyst is found to offer a chiral environment for the reactants. Coordination of the iodine catalyst to the styrene double bond is found to make the benzylic carbon more electrophilic and hence makes it the preferred site for the nucleophilic addition. In the chiral environment of the catalyst, an enhanced polarization of the styrene double bond is noticed when the double bond coordinates through the *si* prochiral face than the *re* face. Nucleophilic addition on the *re* face of the catalyst–substrate complex is associated with a lower activation barrier leading to the experimentally observed *S* enantiomeric product. The stereoselective model developed in this study can be employed to related asymmetric styrene difunctionalizations using similar hypercoordinate iodine catalysts.

## Abstract 2

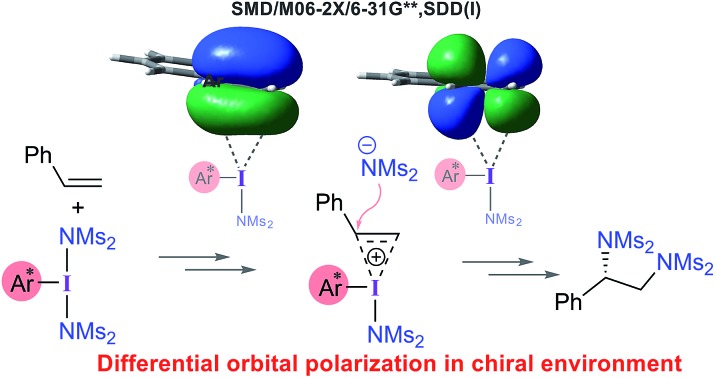
Stereoselectivity in the asymmetric diamination of styrene catalyzed by chiral hypercoordinate iodine originates from the prochiral face recognition when the substrate binds to the catalyst.

## Introduction

Vicinal diamines are important structural elements in pharmaceuticals, natural products, and organo- as well as transition metal catalysts.^1^ Hence, efficient methods for the synthesis of diamines are of great interest.^2^ Palladium- and copper-catalyzed asymmetric diamination of alkenes involving intramolecular C–N bond formation is the most frequently found catalytic diamination reaction.^3^ Among the metal-free approaches, hypercoordinate iodine mediated diamination of alkenes has received considerable attention in recent years.^4^

**Scheme 1:**
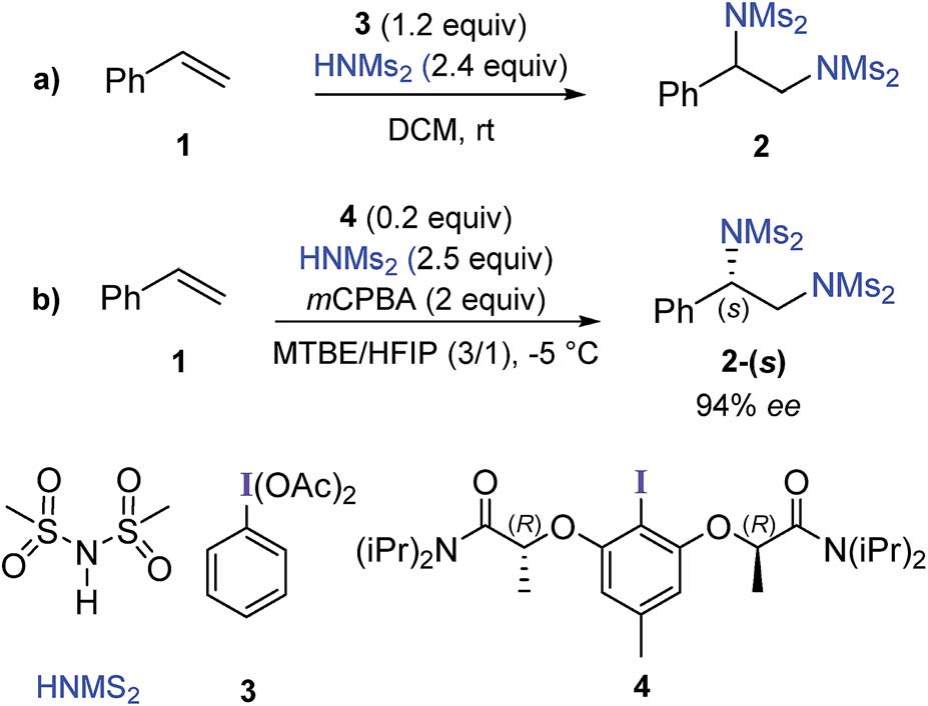
(a) Stoichiometric and (b) catalytic diamination of styrene using hypercoordinate iodine.

Hypercoordinate iodine mediated methods have found widespread acceptance owing to their utility in a diverse set of reactions under milder and environmentally benign conditions.^5^ An excellent strategy for diamination of styrene derivatives in the presence of a stoichiometric amount of phenyliodine(iii) diacetate (PIDA) with two equivalents of sulfonimide HNMs_2_ as the aminating agent (Scheme 1a) was recently reported.^6^ This method offers wide substrate scope and functional group tolerance.^7^ Replacing PIDA with chiral iodoresorcinol derivatives could provide asymmetric diamination.^6^ In a recent catalytic version of this reaction, catalytically active chiral hypercoordinate iodine species was generated *in situ* from the corresponding Ar–I (**4**) and HNMs_2_ in the presence of two equivalents of *meta*-chloroperbenzoic acid (*m*CPBA) as an oxidizing agent (Scheme 1b).^8^

Difunctionalization of alkenes using hypercoordinate iodine(iii) has found interesting applications in the synthesis of complex target molecules.^9^ In particular, iodoresorcinol based catalysts derived from the corresponding Ar–I (similar to **4**) have been recently used for stereoselective difunctionalization of various styrenes, in which the *S* stereogenic center at the benzylic position was formed in the product.9a–c Different chiral iodoresorcinol derivatives differ in terms of the substituents attached to the carbonyl (–N(iPr_2_) in the representative example **4**) and C_*α*_ (–Me in **4**) positions of the chiral arms, while maintaining the *R* configuration at the C_*α*_ position. This observation indicates that the preferred prochiral face of styrene involved in all of these reactions is the same, such that the (*S*)-configured enantiomer of the product is generated. However, the origin of stereoinduction in these reactions is often interpreted using qualitative working hypotheses.^10^ In view of the impressive developments in asymmetric catalysis using hypercoordinate iodine,^11^ we probed the origin of stereoinduction^12^ in styrene diamination by using DFT(SMD_(diethylether)_/M06-2X/6-31G**,SDD(I)) computations.

## Computational methods

All geometry optimizations were performed using the Gaussian09 suite of quantum chemical programs.^13^ The M06-2X density functional was chosen for geometry optimization which accounts for the potentially significant dispersion interactions in relatively larger molecules.^14^ The 6-31G** basis set was chosen for all atoms, except for iodine.^15^ The Stuttgart–Dresden double-zeta valence basis set (SDD) with an effective core potential for 46 inner electrons was employed for iodine.^16^ Solvent effects were incorporated using the universal SMD continuum solvation model developed by Truhlar and Cramer.^17^ The experimental conditions for the catalytic asymmetric reaction employed a solvent system consisting of a 3 : 1 mixture of methyl *tert*-butyl ether (MTBE) and hexafluoroisopropanol. Since the dielectric continuum field for MTBE (*ε* = 4.5) is not available in Gaussian09, implicit solvation calculations for closely related diethyl ether (*ε* = 4.2) were employed. Hexafluoroisopropanol was explicitly included in several stationary points to evaluate specific interactions with the solute. All stationary points were verified by vibrational frequency analysis. Minima (reactants, products, and intermediates) were characterized by having zero imaginary vibrational frequencies, while transition states had only one imaginary frequency. Further verification of the transition states was done using intrinsic reaction coordinate (IRC) calculations in order to connect to the corresponding reactants and products.^18^ We wish to convey that locating various transition state geometries in hypercoordinate iodine catalyzed reactions could be challenging due to geometry convergence issues. Similar computational methods to those employed in this study have successfully been employed in recent studies of hypercoordinate iodine reactions.^19^

Topological analysis of the electron densities, within the Atoms In Molecule (AIM) framework, was carried out using AIM2000 software to identify the bond paths representing the weak inter-atomic interactions.^20^ Natural bond orbital (NBO)^21^ analysis of important stationary points was done to understand important electron delocalizations and charge distributions. To examine the origin of energy difference between the stereocontrolling transition states, the activation-strain model was employed.^22^ In this model, the activation barrier (Δ*E*^‡^) is considered as the sum of (a) destabilizing distortion energies (Δ*E*‡d) in the reactants while going from their ground state geometries to those in the transition states, and (b) stabilizing interaction energy between such deformed reactants (Δ*E*‡i) in the TS geometry. This approach would help quantify the relative distortion and interaction energies between the stereocontrolling transition states.

The discussion is presented on the basis of the Gibbs free energies at 298 K and as obtained at the SMD_(diethylether)_/M06-2X/6-31G**,SDD(I) level of theory. Gibbs free energies are corrected using Truhlar's quasi-harmonic approximation to address the issues arising due to the harmonic oscillator approximation for frequencies lower than 100 cm^–1^.^23^ Gibbs free energies were also calculated using the quasi-rigid rotor harmonic oscillator (RRHO) approximation for the purpose of comparison, which is provided in the ESI.† 24 The energies obtained at the SMD_(diethylether)_/M06-2X/6-31G**,SDD(I) level of theory were refined using a larger basis set using the same functional to learn that the results were similar. Geometries were also optimized at the B3LYP-D3 level of theory. Energy refinements at the B3LYP-D3 level of theory using the geometries at the same level of theory were also carried out to check whether our results were consistent across different functionals.^25^

## Results and discussion

**Scheme 2:**
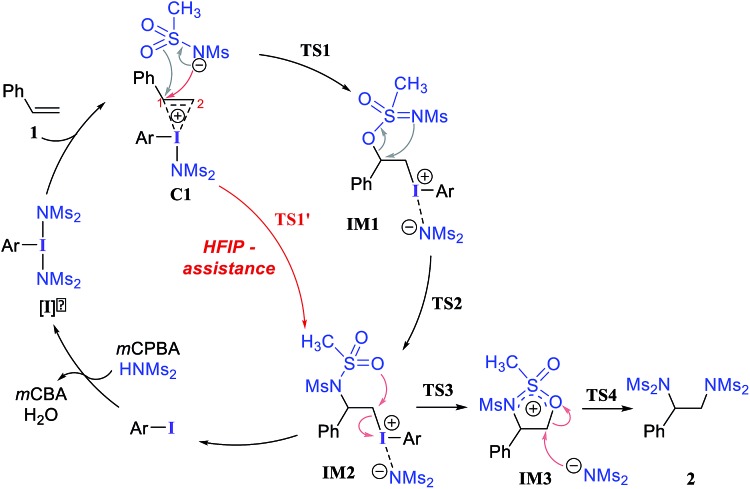
Mechanism for catalytic vicinal diamination of styrene using hypercoordinate iodine.

We have investigated all of the likely elementary mechanistic steps to develop an improved understanding of the key intermediates and transition states (TSs) involved in the diamination reaction, as shown in Scheme 2. An achiral hypercoordinate iodine species (**3′**) was considered first to develop some basic understanding about the key mechanistic features. The factors responsible for stereoinduction are then delineated by considering the chiral hypercoordinate iodine species that is derived from **4**.

First, an achiral PhI(NMs_2_)_2_ (**3′**) is employed as an active catalyst **[I]′** to gather important mechanistic details (Scheme 2).^26^,^27^ The substrate styrene can displace one of the labile imidate (NMs_2_^–^) ligands to form a catalyst–substrate ion-pair complex, **C1**.^28^ The natural bond orbital analysis (NBO) of **C1** revealed a prominent π electron delocalization (45.1 kcal mol^–1^) from the styrene double bond to the I(iii)–N antibonding orbital.^29^ The coordination of the styrenyl double bond to the electrophilic I(iii) center is found to induce a polarization such that the benzylic carbon C_1_ becomes relatively more positive (natural charges on C_1_ = –0.12 and on C_2_ = –0.54 in **C1** whereas that on an unbound free styrene is C_2_ = –0.43 and C_1_ = –0.25), making the C_1_ benzylic carbon more prone to nucleophilic addition. The polarization of the styrene π orbital in this catalyst–substrate complex is evident from a relatively higher π orbital coefficient (0.75) on the C_2_ carbon than on C_1_ (0.65), which was the same for a free styrene (orbital coefficient of 0.70 on both C_1_ and C_2_).

**Fig. 1:**
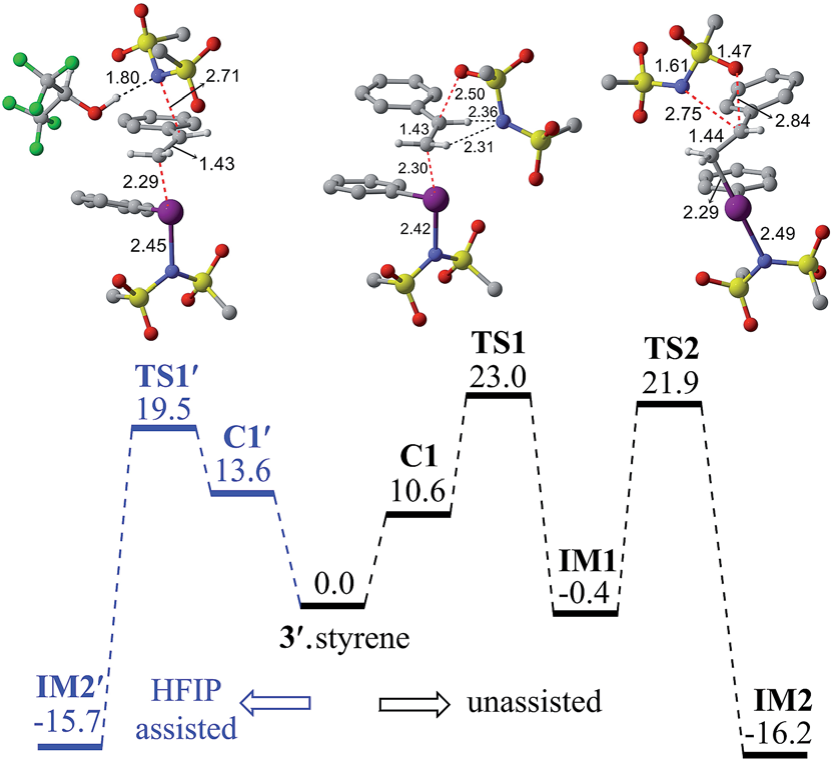
Gibbs free energy (in kcal mol^–1^) profile obtained at the SMD_(diethylether)_/M06-2X/6-31G**,SDD(I) level of theory for the HFIP assisted (left, in blue color) and unassisted (right) iodonium ion formation. Optimized geometries of **TS1′**, **TS1** and **TS2** are shown. All distances are in Å.

In the next step, we considered a rebound mechanism wherein the displaced sulfonamide anion adds to the polarized styrenyl double bond. Due to effective charge delocalization from nitrogen to oxygen in the imidate ion, the nucleophilic addition can occur either through the oxygen or through the nitrogen. The natural charges on the nitrogen (–1.32) and the oxygen (–1.04) of the imidate indicate that a nucleophilic addition through the nitrogen is more likely. However, attempts to locate a transition state for the nucleophilic addition through the nitrogen remained unsuccessful, and instead resulted in an addition through the oxygen (**TS1**).^30^,^31^ The comparison of nucleophilicity indices of 0.61 and 0.08, respectively, for the nitrogen and oxygen suggests that a nucleophilic addition through the softer sulfonyl oxygen is likely to be more preferred at the benzylic carbon of the iodine-bound styrene resulting in the iodonium ion intermediate **IM1**.^32^ In the next step, a concerted intramolecular migration converts **IM1** to **IM2***via***TS2**.^33^ Formation of the second iodonium ion intermediate **IM2** is exergonic by ∼16.0 kcal mol^–1^ (Fig. 1). An intramolecular nucleophilic addition by the sulfonyl oxygen at C_2_ can now provide a cyclic oxazolidine oxide intermediate, **IM3**. The barrier to this elementary step for the expulsion of PhI is 11.4 kcal mol^–1^.^34^ A subsequent nucleophilic ring opening by imidate can furnish the vicinal diamine product **2**. Interestingly, an intermediate with a C_1_–O bond similar to **IM1** was observed during the oxidation of 1,1-diphenyl ethylene using PIDA in the absence of HFIP.^30^

**Fig. 2:**
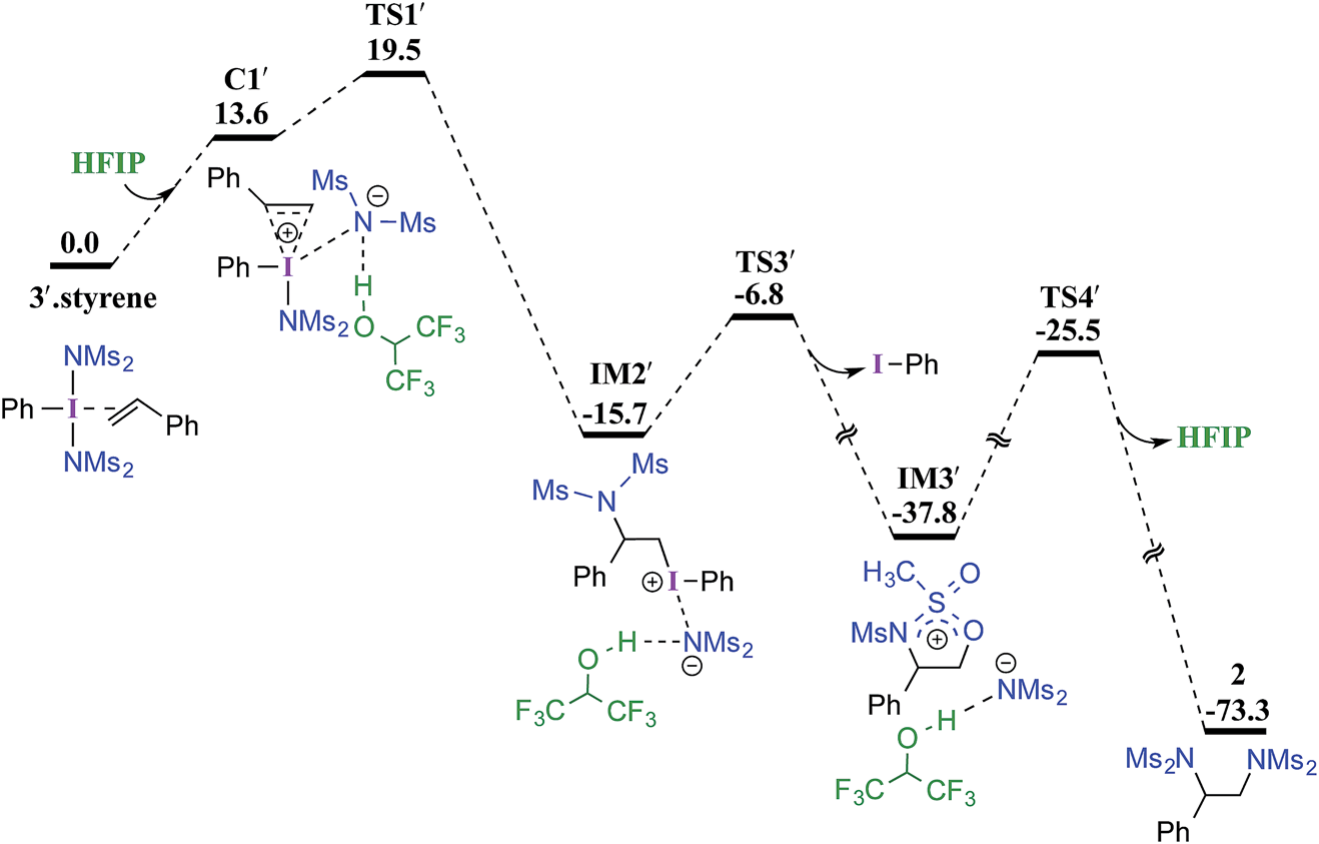
Gibbs free energy (in kcal mol^–1^) profile calculated at the SMD_(diethylether)_/M06-2X/6-31G**,SDD(I) level of theory for the HFIP assisted diamination of styrene.

Earlier reports suggested that fluorinated solvents such as hexafluoroisopropanol (HFIP) are more beneficial in hypercoordinate iodine mediated reactions.^35^ For example, the improved reactivity of PIDA in the presence of HFIP is attributed to hydrogen bonding between the ligands on PIDA and HFIP^36^ which makes the ligands more labile.^37^ We have considered a similar scenario wherein the displaced imidate nitrogen is hydrogen bonded in **C1′** as shown in Fig. 2. Interestingly, the ensuing nucleophilic addition (**TS1′**) on the styrenyl carbon is noted to occur through the imidate nitrogen. The natural charge on the HFIP-bound imidate nitrogen is higher (–1.34) than that on the unbound (–1.32). However, the nucleophilicity index of the HFIP-bound nitrogen is lower (0.58) than that of the unbound (0.61), indicating relatively softer nitrogen in the former case. Thus, explicit involvement of HFIP enables direct formation of the N–C bond in **C1′** leading to **IM2′**. An alternative nucleophilic addition through the imidate oxygen is found to be 4.0 kcal mol^–1^ higher in energy when imidate is associated with HFIP. The HFIP-assisted nucleophilic addition exhibits a barrier of 19.5 kcal mol^–1^, which is 3.5 kcal mol^–1^ lower than that of the unassisted nucleophilic addition (Fig. 1).^38^ Having understood that the HFIP-assisted pathway is energetically preferable, we calculated the energetics for the HFIP-assisted formation of diamine product **2** as shown in Fig. 2.^39^

After shedding light on the mechanism of vicinal diamination of styrene using PhI(NMs_2_)_2_, we have extended these insights to an asymmetric diamination catalyzed by *C*_2_ symmetric iodoresorcinol based chiral catalysts to establish the factors controlling enantioselectivity. As described earlier, nucleophilic addition at the benzylic carbon (**TS1′**) is more preferred than at the terminal carbon of the catalyst–substrate complex **C1′**. The stereochemical outcome of the reaction will therefore be dictated by which of the prochiral faces of styrene binds to the iodine center. It is likely that the chiral environment offered by the catalyst could make the binding through one prochiral face energetically more preferred over the other in the ion-pair catalyst–substrate complex **C1′**. In other words, the efficiency of this prochiral face recognition is expected to impact on the extent of enantioselectivity.

**Fig. 3:**
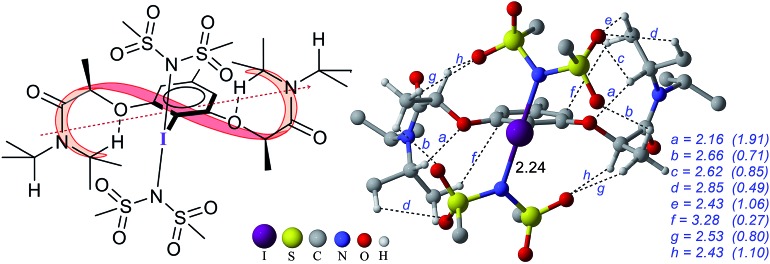
Optimized geometry of catalytically active species **4′**. All distances are in Å and relative energies are in kcal mol^–1^. Important noncovalent interactions (*a–h*) are shown in blue with the corresponding electron densities (*ρ* × 10^–2^ au in parentheses) at the bond critical points along the bond paths. Only selected hydrogen atoms are shown for improved clarity. The helical axis (red dotted line) formed due to the folding of the chiral side chain (ribbon-like depiction) is shown in the structure on the left.

The process of stereoinduction is investigated by examining the stereoelectronic features of the *in situ* generated catalytically active hypercoordinate iodine species **4′**. In the most preferred conformer of the *C*_2_-symmetric **4′**, the chiral arms fold in a right-handed *P* helical fashion and generate a chiral environment around the iodine. The (*R*)-configured stereogenic center at the C_*α*_ is the source of stereoinduction, which facilitates the fold with right-handed *P* helicity. The corresponding *M* helical fold is found to be of 8.0 kcal mol^–1^ higher energy.^12^,^40^ A series of weak non-covalent interactions (NCIs) between the imidate ligands and the chiral arms of the resorcinol backbone (Fig. 3) are found to arrange the chiral arms in a helical assembly. This folded assembly of the catalyst provides an asymmetric environment for the incoming styrene. How the binding of the *si* and *re* prochiral faces of styrene to the iodine is recognized differently in the catalyst–substrate complexes is analyzed using the cationic complexes (**C1^+^**) devoid of the displaced nucleophile imidate associated with HFIP.^41^ It is noticed that the catalyst–substrate complex **C1*_si_*^+^** wherein styrene interacts through its *si* face is lower by 1.8 kcal mol^–1^ than the corresponding *re* face binding (**C1*_re_*^+^**).

**Fig. 4:**
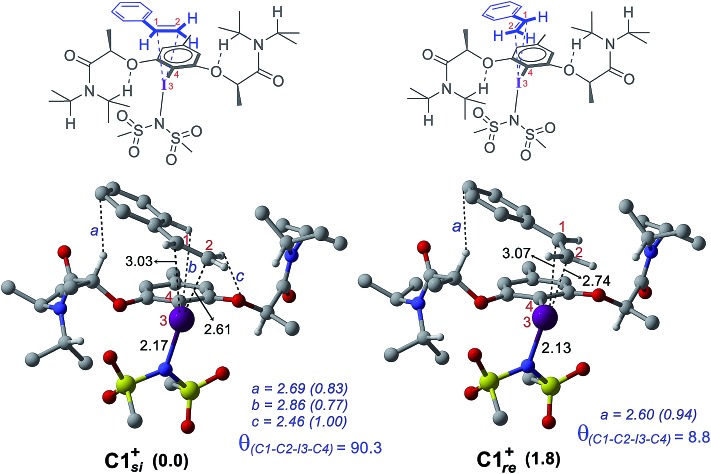
Optimized geometries of cationic catalyst–substrate complexes **C1*_si_^+^*** and **C1*_re_^+^*** as well as the corresponding qualitative representations. All distances are in Å and relative energies are in kcal mol^–1^. Important noncovalent interactions (*a–c*) are shown in blue with the corresponding electron densities (*ρ* × 10^–2^ au in parentheses) at the bond critical points along the bond paths. Only selected hydrogen atoms are shown for improved clarity.

The differential recognition of the styrenyl prochiral faces can be understood as follows. In the *si* face catalyst–substrate complex, styrene is dispositioned closer to the iodine center, as evident from the shorter C_2_–I distance (2.61 Å) than in the *re* complex (2.74 Å), indicating enhanced catalyst–substrate interaction (Fig. 4). Further, the styrene double bond is noted to remain staggered and orthogonal with respect to the C_4_–I_3_ bond of the catalyst (*θ*_(C1–C2–I3–C4)_ = 90.3°) in **C1*_si_^+^*** whereas it is eclipsed in **C1*_re_^+^*** (*θ*_(C1–C2–I3–C4)_ = 8.8°).^42^ The arrangement of styrene in these catalyst–substrate complexes facilitates a C–H···π interaction between the C_*α*_–H of the chiral side chain and the phenyl group of styrene (shown as *a* in Fig. 4). A larger deviation from the eclipsed alignment of the styrene double bond in the *re* complex (measured in terms of dihedral *θ*_(C1–C2–I3–C4)_) will result in loss of this critical interaction. Additional weak noncovalent interactions are noticed in the *si* mode of binding, which are absent in the *re* complex. Natural bond orbital (NBO) analysis revealed an enhanced π electron delocalization from the styrene double bond to the I–N antibonding orbital in **C1*_si_*^+^** (46.1 kcal mol^–1^) compared to that in **C1*_re_*^+^** (30.7 kcal mol^–1^). Comparison of a free styrene with a bound styrene as noted in the catalyst–substrate complexes is carried out to understand the effect of iodine coordination on the reactivity and selectivity.

**Fig. 5:**
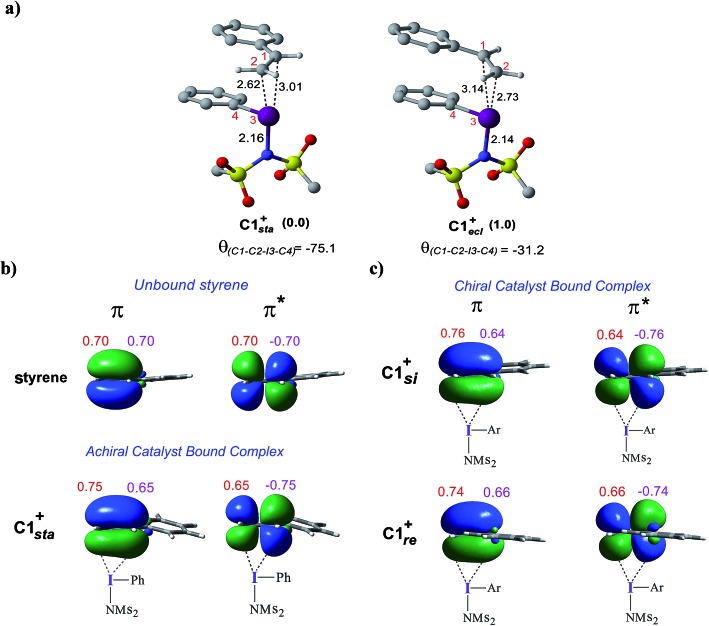
(a) Optimized geometries of **C1*_sta_*^+^** and **C1*_ecl_*^+^**. (b) Important natural bond orbitals (NBOs) corresponding to the π and π* of (b) a free styrene as well as those in the iodine achiral catalyst–substrate complex **C1*_sta_*^+^**, and (c) in the chiral catalyst–substrate complexes. Orbital coefficients on C_1_ (in purple) and C_2_ (in red) carbon atoms are given for each NBO.

To examine how the relative orientation of the styrene double bond and the Ar–I bond influences their energies, catalyst–substrate complexes with staggered and eclipsed orientations of the substrate are analyzed for an achiral catalyst (Fig. 5a). The staggered arrangement (**C1*_sta_*^+^**) is found to be 1.0 kcal mol^–1^ lower in energy than the eclipsed geometry (**C1*_ecl_*^+^**). In the case of the achiral catalyst, binding of both the prochiral faces of styrene is found to be energetically similar, suggesting no facial discrimination.

To gather more insights, the π and π* natural bond orbitals (NBOs) in a free styrene as well as those in an iodine-bound styrene are analyzed. These are critical NBOs, as the donor π and acceptor π* are, respectively, involved in the catalyst–substrate complex formation and in the nucleophilic addition. Both benzylic (C_1_) and terminal carbon (C_2_) atoms of the free styrene contribute equally to the π orbital as evident from the corresponding orbital coefficients (C_1_ = C_2_ = 0.70, Fig. 5b). Interestingly, in the catalyst–substrate complexes, the π orbital is polarized toward the terminal carbon as indicated by a higher orbital coefficient on C_2_ than that on C_1_ whereas the corresponding π* orbital is more polarized toward the benzylic carbon C_1_. In the achiral catalyst–substrate complexes, the π orbital polarization is noted to be higher in the case of the staggered geometry of styrene in **C1*_sta_*^+^** (coefficients on C_1_ and C_2_ are 0.65 and 0.75, respectively) compared to that in **C1*_ecl_*^+^** (C_1_ = 0.64 and C_2_ = 0.76).^43^ More interestingly, a differential polarization of the π and π* is noted in the chiral catalyst–substrate complexes depending on the prochiral face through which styrene binds to the iodine center. The styrene double bond in the *si* binding mode, which has a staggered arrangement, is found to be more polarized than in the *re* complex (Fig. 5c). The orbital coefficients for filled π in **C1*_si_*^+^** are C_1_ = 0.64 and C_2_ = 0.76 and those in **C1*_re_*^+^** are C_1_ = 0.66 and C_2_ = 0.74. The chirality induced differential polarization of the styrenyl double bond, as described here, can have a pronounced impact on the prochiral face recognition and hence the stereochemical course of the reaction. Akin to the orbital polarization noted here, the natural charge on C_1_ and C_2_ exhibited similar features.^43^ In summary, the enhanced polarization of the styrene π orbitals, particularly that of the π* orbital in the lower energy staggered catalyst–substrate complex, improves the propensity toward the nucleophilic addition of the imidate ion.

**Fig. 6:**
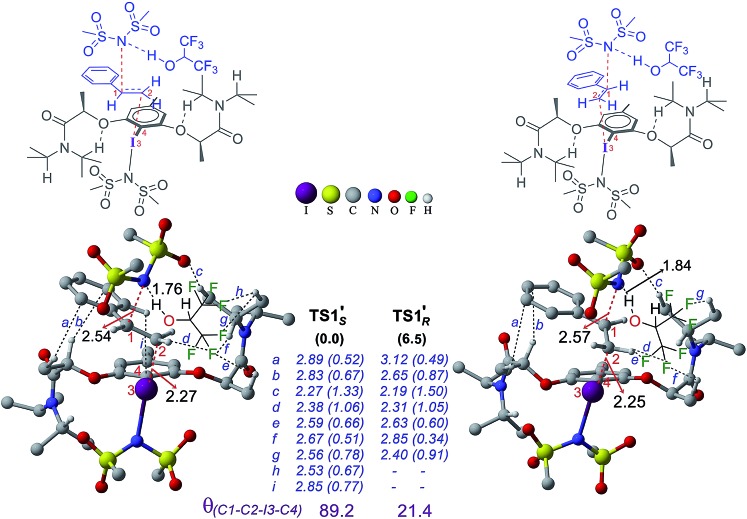
Optimized geometries of the stereoselective transition states **TS1′*_S_*** and **TS1′*_R_***. Relative Gibbs free energy is given in parentheses. Important noncovalent interactions (*a–i*) are shown in blue along with the corresponding electron densities (*ρ* × 10^–2^ au in parentheses) at the bond critical points along the bond paths. All distances are in Å.

In the next step, stereoselective addition of the imidate on the benzylic position of styrene bound to chiral catalyst **4′** is considered (for the geometry of the catalyst, see Fig. 3). The C–N bond formation *via***TS1′** leads to the formation of an iodonium ion intermediate (**IM2′**). Nucleophilic addition of the imidate to the catalyst–substrate complexes **C1′*_si_*** and **C1′*_re_*** is separately examined.^44^ The transition state for the imidate addition to the open *re* face of the bound styrene in **C1′*_si_*** is denoted as **TS1′*_S_*** as the new stereogenic center created in this mode would be of *S* configuration. The relative Gibbs energy of **TS1′*_S_*** is found to be 6.5 kcal mol^–1^ lower in energy than that of **TS1′*_R_***. Similarly, the elementary step barrier for the nucleophilic addition through **TS1′*_S_*** is 3.1 kcal mol^–1^ lower than that for **TS1′*_R_***. A staggered arrangement of the styrene double bond (*θ*_(C1–C2–I3–C4)_ = 89.2°) with respect to the Ar–I bond is noted in the lower energy **TS1′*_S_*** whereas it remains in an eclipsed (*θ*_(C1–C2–I3–C4)_ = 21.4°) geometry in the higher energy **TS1′*_R_***.^45^ Analysis of TSs shows the presence of certain types of noncovalent interactions (denoted as *h* and *i* in Fig. 6) between the reactants (styrene and imidate) and the catalyst in the case of **TS1′*_S_*** which are absent in **TS1′*_R_***. However, these weakly differentiating interactions are rather inadequate toward rationalizing an overwhelming preference for imidate addition *via***TS1′*_S_***. Hence, we have performed activation-strain analysis on these diastereomeric TSs by fragmenting them into three parts such as the catalyst, styrene and nucleophile. All three fragments in **TS1′*_R_*** exhibited relatively higher distortion energy than in **TS1′*_S_*** – the sum of which amounts to 14.4 kcal mol^–1^ in the former.^46^ The stabilizing interaction energy between these three fragments is also higher (–6.9 kcal mol^–1^) in **TS1′*_R_***. On balance, the distortion appears to be a key contributor of the energy difference between the diastereomeric TSs. The difference in the elementary step barriers for the nucleophilic addition of the imidate on the *re* face of the catalyst–substrate complex and that on the *si* face is found to be 3.1 kcal mol^–1^, exhibiting a preference toward the formation of the *S* enantiomeric product.^47^ While the difference in barriers corresponds to an ee of 98% which is in good agreement with the experimental observation of 94%, the relative energies of the diastereomeric transition states result in a modest overestimation of ee.

## Conclusions

The energetically favorable mechanism and the origin of enantioselectivity in hypercoordinate iodine catalyzed vicinal diamination of styrene have been identified. Styrene coordination to the electrophilic iodine center polarizes the double bond making the benzylic carbon susceptible to nucleophilic addition by imidate derived from the active catalyst ArI(NMs_2_)_2_. Hexafluoroisopropanol-assisted nucleophilic addition for the C–N bond formation proceeds with a lower energy barrier and results in an iodonium ion intermediate. Under the chiral environment provided by the iodoresorcinol catalyst, the coordination of the *si* prochiral face of styrene is more preferred due to the relatively more effective catalyst–substrate interaction than in the *re* face complex. This prochiral face recognition is found to have a direct impact on the enantioselectivity of this catalytic asymmetric reaction. The kinetic advantage for nucleophilic addition of the imidate is more for the *si*-face of the catalyst–substrate complex, which could be traced to a relatively lower distortion in the reactant partners in the stereocontrolling transition state. The computed % ee is in good agreement with the experimental observation.

## Conflicts of interest

The authors declare no competing financial interest.

## Supplementary Material

Supplementary information
