# Supplementary material for: Hypercoordinate iodine for catalytic asymmetric diamination of styrene: insights into the mechanism, role of solvent, and stereoinduction
Source: Chem Sci. 2019 Jun 10;10(29):7082–90. doi: 10.1039/c9sc01513b (PMC6676474; doi:10.1039/c9sc01513b)
Supplement: Supplementary file 1 [file SC-010-C9SC01513B-s001.pdf]

Electronic Supplementary Material (ESI) for Chemical Science.
This journal is © The Royal Society of Chemistry 2019

Supporting Information

Hypercoordinate Iodine for Catalytic Asymmetric Diamination of Styrene: Insights into Mechanism, Role of Solvent, and Stereoinduction

A. Sreenithya,[a] Christopher M. Hadad,[b] and Raghavan B. Sunoj[a,*]

[a]Department of Chemistry, Indian Institute of Technology Bombay, Powai, Mumbai 400076, India

[b]Department of Chemistry and Biochemistry, The Ohio State University, 100 West 18th Avenue, Columbus, Ohio 43210, USA

e-mail: sunoj@chem.iitb.ac.in
Telephone: +91-222-576-7173
Fax: +91-222-576-7152

Table of Contents

**List of Schemes**		
Scheme S1	Transformation of $PhI(OAc)_2$ to $PhI(NMs_2)_2$	S4
Scheme S2	Possible pathways for the conversion of **IM2'** to product **2**	S7
Scheme S3	Formation of diaminated product $\mathbf{2}_{S,R}$ from $\mathbf{C1}_{si\text{-}re}$**'**	S17
**List of Figures**		
Fig. S1	Optimized geometries of **3'**, **3'…**styrene and **C1**	S4
Fig. S2	Gibbs free energy profile calculated for the unassisted diamination proceeding through intramolecular 1,3-migration	S5
Fig. S3	Gibbs free energy profile calculated for the unassisted diamination proceeding through intramolecular nucleophilic addition	S6
Fig. S4	NPA charges on the free and HFIP bound imidate	S6
Fig. S5	Gibbs free energy profile for the conversion of **IM2'** to product through different mechanistic pathways	S7
Fig. S6	Optimized geometries of **TS3'**, $\mathbf{TS3'}_{SN2}$ and $\mathbf{TS3'}_{azi}$.	S8
Fig. S7	Optimized geometries of HFIP assisted nucleophilic addition through imidate nitrogen and imidate oxygen	S8
Fig. S8	Optimized geometries of *P* and *M* helical assembly of active species **4'**	S8
Fig. S9	Important conformers of active species **4'** with *P* helical assembly	S9
Fig. S10	Optimized geometries of the chiral catalyst-substrate complexes	S14
Fig. S11	Nucleophilic addition transition states with ($\mathbf{TS1'}_{si}$) and without HFIP ($\mathbf{TS1}_{si}$) assistance in the chiral environment	S14
Fig. S12	Optimized geometries of diastereomeric catalyst-substrate complexes for **S2**	S16
**List of Tables**		
Table S1	Comparison of Gibbs free energies corrected with quasi-harmonic approximation and quasi rigid rotor harmonic oscillator (RRHO) approximation	S10
Table S2	Comparison of relative Gibbs free energies calculated at the M06-2X level of theory	S11
Table S3	Comparison of relative Gibbs free energies at the **L1** and **L5** levels of theory	S11
Table S4	Total electronic energies and the BSSE corrected energies at the **L6** level of theory	S12
Table S5	Comparison of relative Gibbs free energies calculated at the B3LYP-D3 level of theory	S13
Table S6	Summary of natural bond orbital analysis on free styrene and the iodine bound styrene in cationic catalyst-substrate complexes	S13
Table S7	Distortion and interaction energies of diastereomeric TSs	S15

Table S8	Comparison of relative Gibbs free energies for chiral complexes and transition states calculated at different levels of theories	S15
Cartesian coordinates for important stationary points		S18

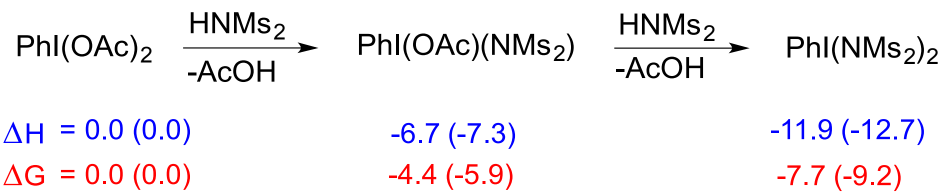

Scheme S1. Sequential transformation of $PhI(OAc)_2$ to $PhI(NMs_2)_2$. Relative energies (kcal/mol) of different species at the $SMD_{(diethylether)}$/M06-2X/6-31G**,SDD(I) level of theory are provided. Energies in dichloromethane as the solvent continuum obtained at the $SMD_{(DCM)}$/M06-2X/6-31G**,SDD(I) level of theory are given in parentheses.

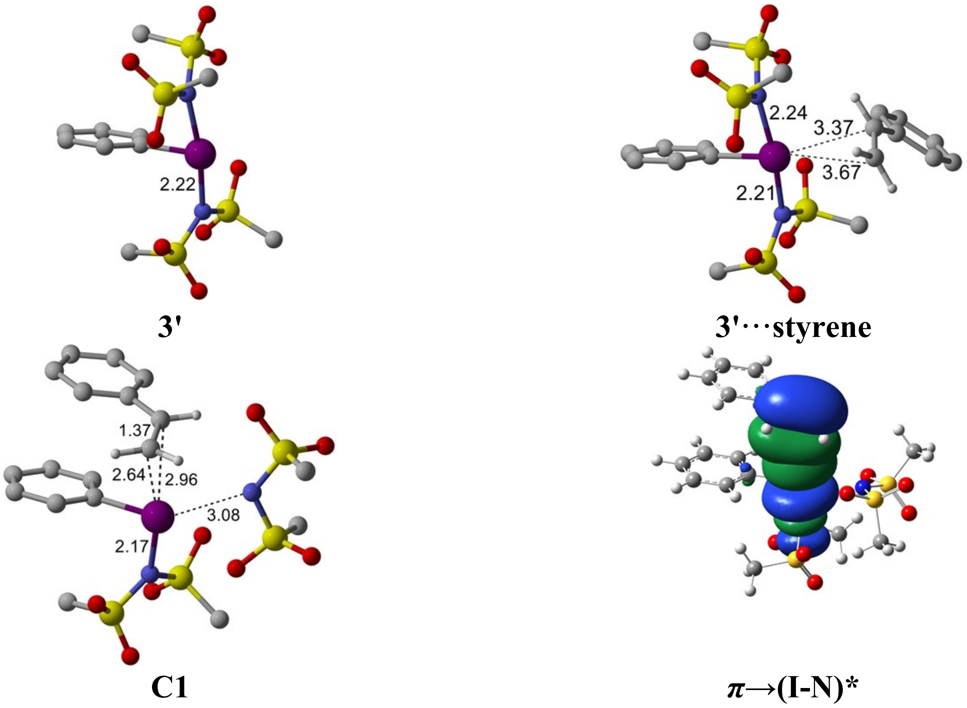

Fig. S1 Optimized geometries of **3'**, **3'···styrene** and **C1**. The π→(I-N)* delocalization in **C1** is shown using NBO. All distances are in Å.

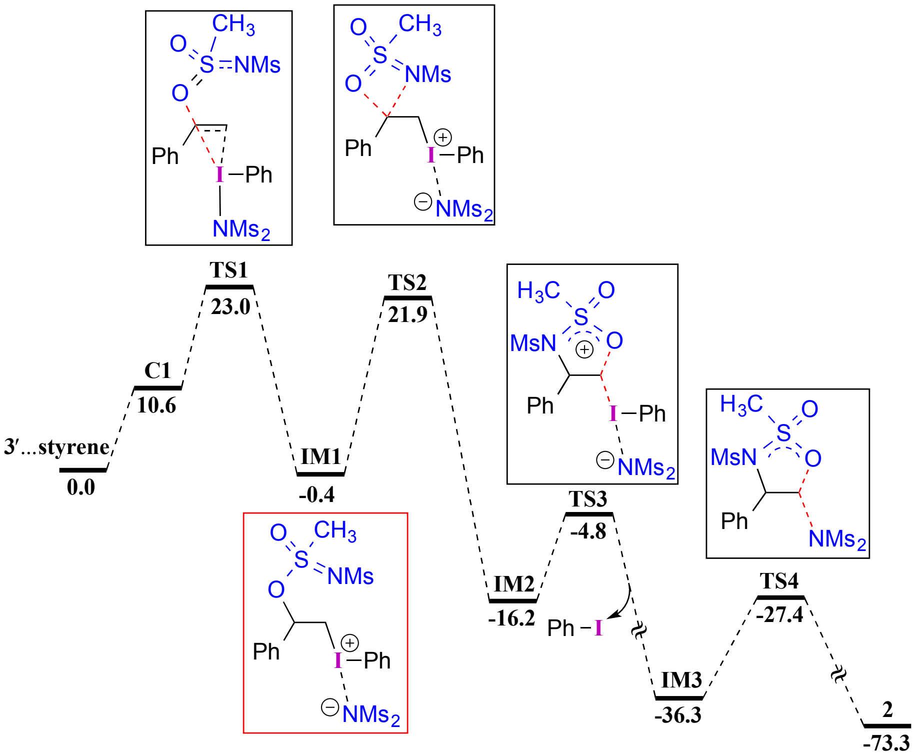

Fig. S2 Gibbs free energy (in kcal/mol) profile calculated at the $SMD_{(diethylether)}$/M06-2X/6-31G**,SDD(I) for styrene diamination catalyzed by hypercoordinate iodine in the unassisted pathway (without explicit HFIP) where **IM1** proceeds through an intramolecular 1,3-migration (**TS2**).

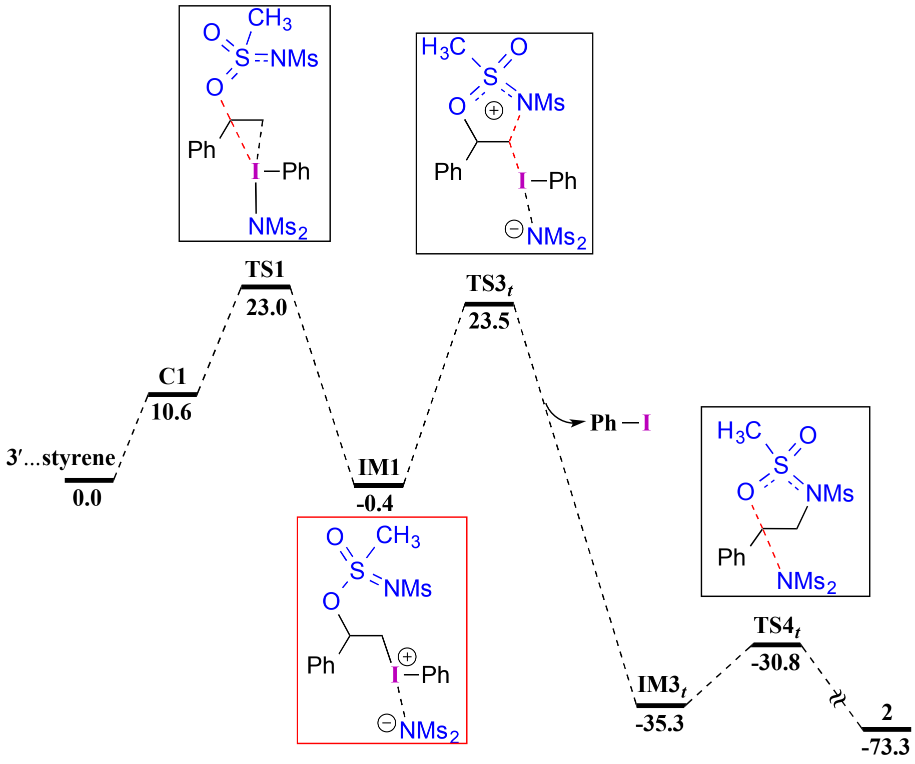

Fig. S3 Gibbs free energy (in kcal/mol) profile calculated at the SMD$_{(diethylether)}$/M06-2X/6-31G**,SDD(I) for styrene diamination catalyzed by hypercoordinate iodine in the unassisted pathway (without explicit HFIP) where **IM1** proceeds through an intramolecular nucleophilic addition at the terminal carbon C_1 (**TS3$_t$**).

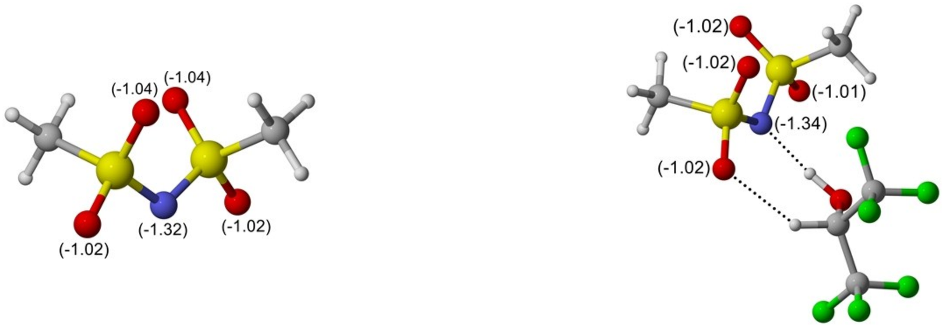

Fig. S4 NPA charges of free imidate and HFIP bound imidate.

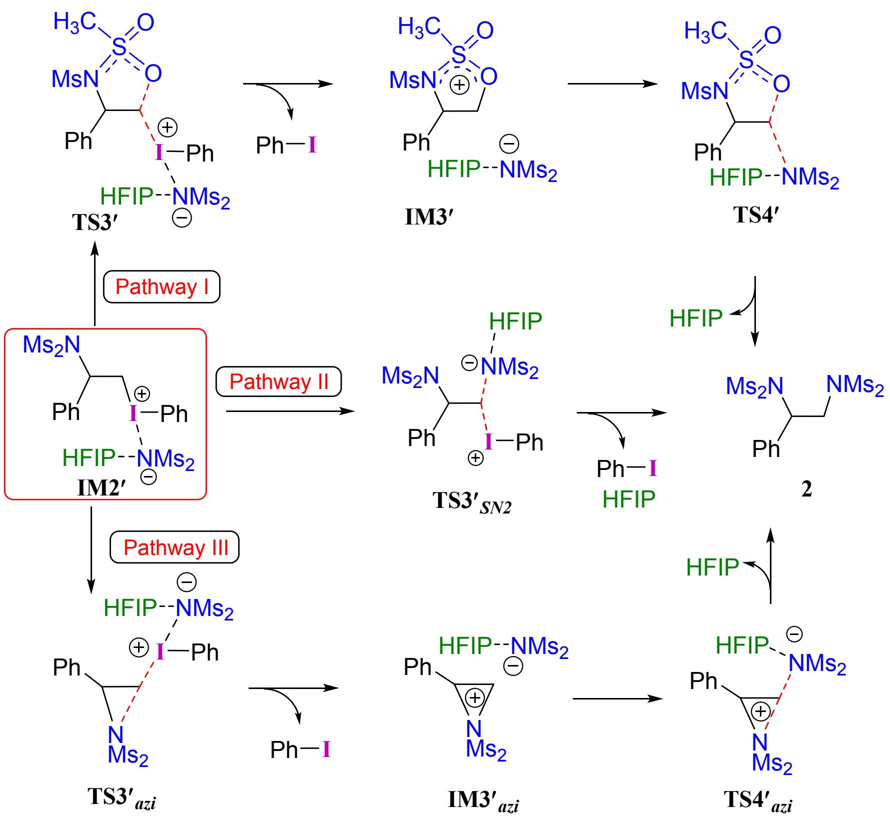

Pathway I : oxazolidine oxide pathway
Pathway II : S_N2 pathway
Pathway III: aziridinium pathway

Scheme S2 Different mechanistic possibilities for the conversion of iodonium ion intermediate **IM2'** to diamine product **2**.

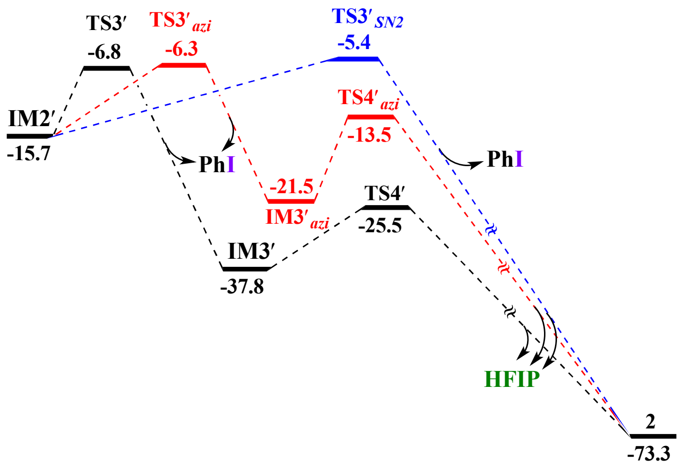

Fig. S5 Energies associated with different possible transformations of **IM2'** to product **2**.

Pathways I and III can be regarded competitive for the C-N bond formation.

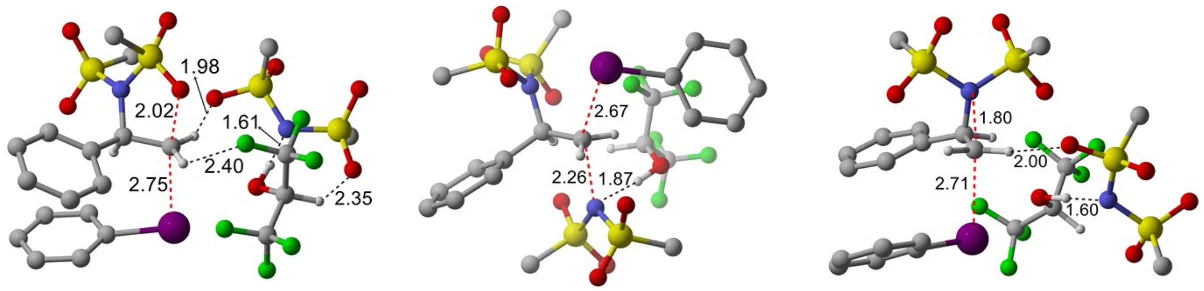

TS3' **TS3'** $_{SN2}$ **TS3'** $_{azi}$

Fig. S6 Optimized geometries of **TS3'**, **TS3'** $_{SN2}$ and **TS3'** $_{azi}$. All distances are in Å.

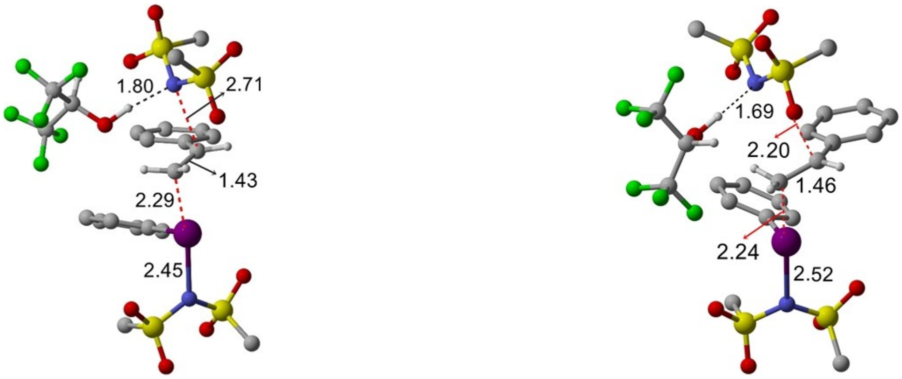

TS1' (0.0) **TS1'** $_{o}$ (4.0)

Fig. S7 Optimized geometries of HFIP assisted nucleophilic addition through imidate nitrogen (**TS1'**) and imidate oxygen (**TS1** $_{o}$ **'**). Relative Gibbs free energies (in kcal/mol) are given in parentheses. All distances are in Å.

a) **b)**

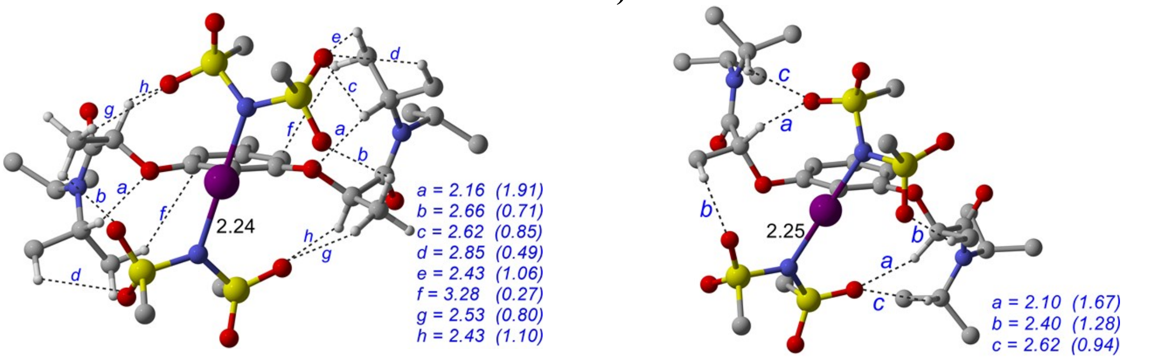

4' $_{P}$ (0.0) **4'** $_{M}$ (8.9)

Fig. S8 Optimized geometries of a) *P* and b) *M* helical assembly of active species **4'**. Relative Gibbs free energy (kcal/mol) is given in parenthesis. Various types of intramolecular

noncovalent interactions are shown (using letters *a* – *h*) with the corresponding electron densities (ρ × 10^{-2} a.u in parentheses) at the bond critical points along the bond paths. All distances are in Å.

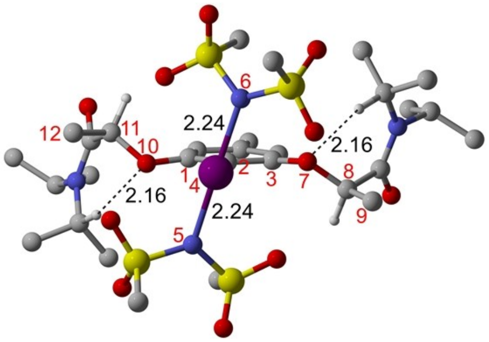

$\theta_{(3\text{-}2\text{-}4\text{-}6)}$ = 72.5, $\theta_{(1\text{-}2\text{-}4\text{-}5)}$ = 72.1
$\theta_{(3\text{-}7\text{-}8\text{-}9)}$ = -157.9, $\theta_{(1\text{-}10\text{-}11\text{-}12)}$ = -157.9
4' (0.0)

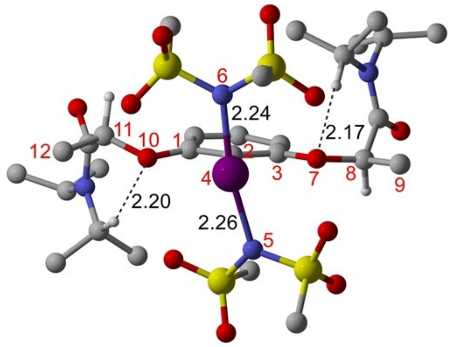

$\theta_{(3\text{-}2\text{-}4\text{-}6)}$ = 104.8, $\theta_{(1\text{-}2\text{-}4\text{-}5)}$ = 106.6
$\theta_{(3\text{-}7\text{-}8\text{-}9)}$ = 173.6, $\theta_{(1\text{-}10\text{-}11\text{-}12)}$ = 173.6
4'-conf1 (6.5)

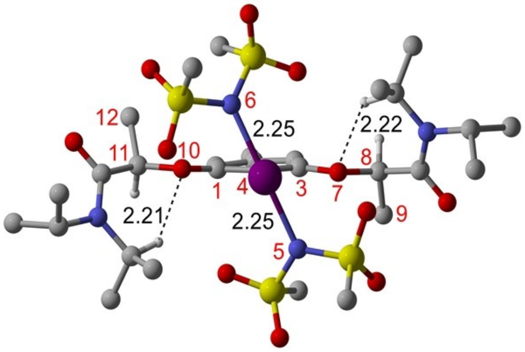

$\theta_{(3\text{-}2\text{-}4\text{-}6)}$ = 115.2, $\theta_{(1\text{-}2\text{-}4\text{-}5)}$ = 115.2
$\theta_{(3\text{-}7\text{-}8\text{-}9)}$ = -69.2
4'-conf2 (10.5)

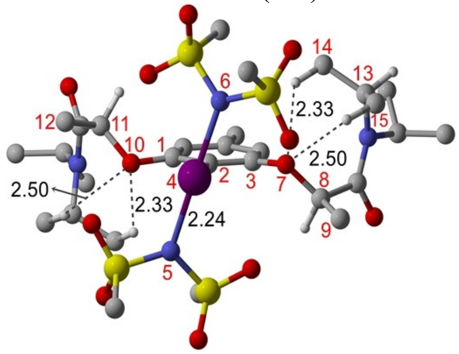

$\theta_{(3\text{-}2\text{-}4\text{-}6)}$ = 70.6, $\theta_{(1\text{-}2\text{-}4\text{-}5)}$ = 70.8
$\theta_{(3\text{-}7\text{-}8\text{-}9)}$ = -149.0, $\theta_{(1\text{-}10\text{-}11\text{-}12)}$ = -148.6
4'-conf3 (17.5)

Fig. S9 Important conformers of the lower energy active species **4'** with *P* helical assembly obtained by varying (i) the dihedral between aryl ring plane and ligands on iodine ($\theta_{3\text{-}2\text{-}4\text{-}6}$ and $\theta_{1\text{-}2\text{-}4\text{-}5}$) and (ii) the orientation of the homobenzylic methyl group ($\theta_{3\text{-}7\text{-}8\text{-}9}$ and $\theta_{1\text{-}10\text{-}11\text{-}12}$). Relative Gibbs free energy (kcal/mol) is given in parenthesis. All distances are in Å and dihedral angles in °.

Table S1 Comparison of Relative Gibbs Free Energies (in kcal/mol) of Important Transition States and Intermediates Involved in Styrene Diamination Obtained at the $SDD_{(diethylether)}$/M06-2X/6-31G**,SDD(I) Level of Theory. The Gibbs Free Energies are Corrected with Refined Entropy Estimates Obtained using Quasi-Harmonic Approximation and Quasi Rigid Rotor Harmonic Oscillator (RRHO) Approximation

Stationary Point	ΔG with Quasi-harmonic correction	ΔG with RRHO correction
**3'···styrene**	0.0	0.0
**C1'**	13.6	13.3
**TS1'**	**19.5**	**19.2**
**IM2'**	-15.7	-16.8
**TS3'**	-6.8	-7.1
**IM3'**	-37.8	-38.1
**TS4'**	-25.5	-25.7
**2**	-73.3	-73.7
**C1**	10.6	10.5
**TS1**	**23.0**	**22.3**
**IM1**	-0.4	-1.0
**TS2**	21.9	21.5
**IM2**	-16.2	-17.2

Table S2 Computed Relative Gibbs Free Energies (in kcal/mol) of Important Transition States and Intermediates Involved in Styrene Diamination at the M06-2X Level of Theory. Energy Refinements are done at the **L2-4**[a] Level of Theory using the Geometries Obtained at the **L1** Level of Theory

Stationary Point	L1	L2	L3	L4
**3'···styrene**	0.0	0.0	0.0	0.0
**C1'**	13.6	14.5	15.0	14.5
**TS1'**	19.5	19.0	20.5	20.5
**IM2'**	-15.7	-12.6	-10.7	-11.1
**TS3'**	-6.8	-1.0	2.8	2.6
**IM3'**	-37.8	-29.1	-26.8	-25.8
**TS4'**	-25.5	-16.5	-13.5	-13.3
**2**	-73.3	-65.0	-61.9	-61.6
**C1**	10.6	10.3	11.0	10.7
**TS1**	23.0	21.0	22.0	22.1
**IM1**	-0.4	1.8	2.0	2.4
**TS2**	21.9	18.6	20.0	20.0
**IM2**	-16.2	-15.1	-13.3	-13.2

[a] L1=SMD$_{(diethylether)}$/M06-2X/6-31G**,SDD(I); L2=SMD$_{(diethylether)}$/M06-2X/6-311+G**,SDD(I); L3 = SMD$_{(diethylether)}$/M06-2X/6-311+G**,Def2TZVP(I); L4 = SMD$_{(diethylether)}$/ M06-2X/6-311++G**,aug-cc-pVTZ-PP(I).

Table S3 Comparison of Relative Gibbs Free Energies (in kcal/mol) of Important Transition States and Intermediates Obtained at the **L1** and **L5** Levels of Theory

Stationary Point	L1	L5
**3'···styrene**	0.0	0.0
**C1'**	13.6	11.5
**TS1'**	19.5	18.2
**IM2'**	-15.7	-2.4
**TS3'**	-6.8	11.4
**IM3'**	-37.8	-5.3
**TS4'**	-25.5	-0.5
**2**	-73.3	-44.3
**C1**	10.6	9.2
**TS1**	23.0	20.9
**IM1**	-0.4	11.6
**TS2**	21.9	18.9
**IM2**	-16.2	-4.5

L1=SDD$_{(diethylether)}$/M06-2X/6-31G**,SDD(I); L5= SDD$_{(diethylether)}$/M06-L/6-311G**,aug-cc-pVTZ-PP

Table S4 Relative Total Electronic Energy (in kcal/mol) for Important Transition States and Intermediates Involved in Styrene Diamination Obtained at the **L6** Level of Theory and the Corresponding Basis Set Superposition Error (BSSE) Corrected Energy.[a]

Stationary Points	L6	$L6_{BSSE}$
**3'···styrene**	0.0	0.0
**C1'**	-3.3	8.1
**TS1'**	8.7	17.1
**IM2'**	-30.5	-23.0
**TS3'**	-26.5	-15.8
**IM3'**	-42.6	-33.5
**TS4'**	-32.9	-23.0
**2**	-63.4	-63.4
**C1**	14.0	16.9
**TS1**	36.4	36.4
**IM1**	4.9	7.5
**TS2**	33.8	34.6
**IM2**	-11.8	-10.9

[a] - BSSE calculations are done at the **L6** (M06-2X/6-31G**,SDD(I)) level of using the Geometries Obtained at the **L1** ($SDD_{(diethylether)}$/M06-2X/6-31G**,SDD(I)) Level of Theory. BSSE corrected energies of all the stationary points involving HFIP are ~9 kcal/mol higher than those without correction. In all other stationary points, BSSE corrected energies are 0-3 kcal/mol higher than those without correction. For BSSE calculations, **3′□□□styrene** is fragmented to two neutral fragments, **3′** and **styrene**. All other stationary points which do not involve HFIP are fragmented in to two where NMs_2^- is taken as fragment 1 and rest of the electrophilic system as fragment 2. Three fragments are considered in stationary points involving HFIP, where the third fragment being the neutral HFIP.

Table S5 Computed Relative Gibbs Free Energies (in kcal/mol) of Important Transition States and Intermediates Involved in Styrene Diamination at the B3LYP-D3 Level of Theory. Energy Refinements are done at the **L8**[a] Level of Theory using the Geometries Obtained at the **L7** Level of Theory

Stationary Point	L7	L8
**3'···styrene**	0.0	0.0
**C1'**	5.5	9.9
**TS1'**	9.1	12.2
**IM2'**	-18.1	-11.3
**TS3'**	-17.0	-7.1
**IM3'**	-38.2	-26.9
**TS4'**	-30.3	-19.5
**2**	-78.3	-60.8
**C1**	10.0	9.5
**TS1**	19.1	16.7
**IM1**	3.1	4.8
**TS2**	17.4	13.8
**IM2**	-12.2	-11.1

[a] **L7**=$SMD_{(diethylether)}$/B3LYP-D3/6-31G**,SDD(I); **L8**=$SMD_{(diethylether)}$/B3LYP-D3/6-311+G**,SDD(I)

Table S6 The π Electron Delocalization Energy (kcal/mol) to (I-N)* Antibonding Orbital, Natural Charges (a.u) and Natural Bond Orbital Coefficients on Alkene Carbons of Free Styrene and the Bound Styrene in Various Catalyst-Substrate Complexes

	**styrene**	$\mathbf{C1}_{sta}^{+}$	$\mathbf{C1}_{ecl}^{+}$	$\mathbf{C1}_{si}^{+}$	$\mathbf{C1}_{re}^{+}$
$\pi_{(C1\text{-}C2)}$→(I-N)*	-	44.9	31.0	46.1	30.7
NPA on C_1	-0.25	-0.10	-0.13	-0.09	-0.14
NPA on C2	-0.43	-0.57	-0.55	-0.58	-0.54
Coefficient of $\pi_{(C1\text{-}C2)}$ on C_1	0.70	0.65	0.66	0.64	0.66
Coefficient of $\pi_{(C1\text{-}C2)}$ on C_2	0.70	0.75	0.74	0.76	0.74
Coefficient of $\pi^*_{(C1\text{-}C2)}$ on C_1	-0.70	-0.75	-0.74	-0.76	-0.74
Coefficient of $\pi^*_{(C1\text{-}C2)}$ on C_2	0.70	0.65	0.66	0.64	0.66

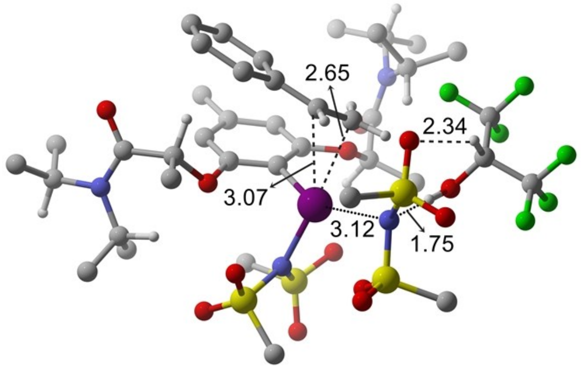

C1'$_{si}$ (0.0)

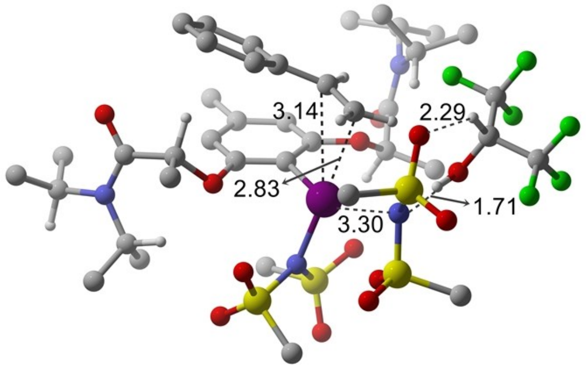

C1'$_{re}$ (3.4)

Fig. S10 Optimized geometries of the chiral catalyst-substrate complexes **C1'$_{si}$** and **C1'$_{re}$**. Relative Gibbs Free Energies (in kcal/mol) are given in parentheses. All distances are in Å.

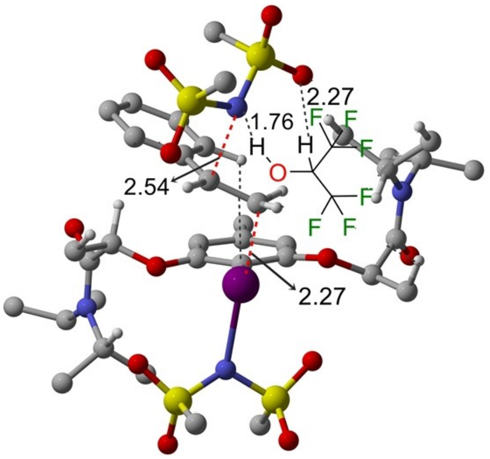

TS1'$_{S}$ (0.0)

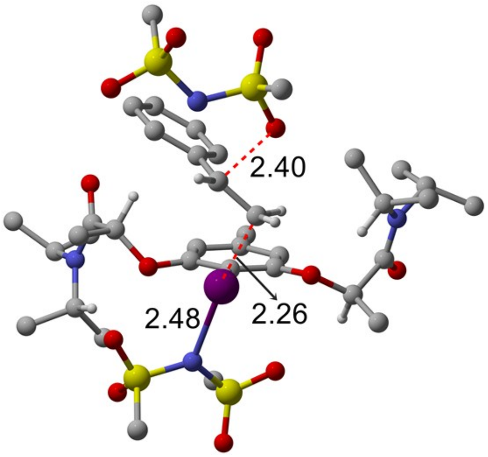

TS1$_{S}$ (8.9)

Fig. S11 Optimized geometries of the nucleophilic addition transition states with (**TS1'$_{S}$**) and without HFIP (**TS1$_{S}$**) assistance in the chiral environment. Relative Gibbs Free Energies (in kcal/mol) are given in parentheses. Nucleophilic addition occurs through the imidate nitrogen in **TS1'$_{S}$** while it is through imidate oxygen in **TS1$_{S}$**. All distances are in Å.

Table S7 The Relative Distortion ($\Delta\Delta E_d^{\ddagger}$) and the Total Interaction Energies ($\Delta\Delta E_i^{\ddagger}$) (in kcal/mol) of Diastereomeric TSs [a]

	Fragment	**TS1'$_S$**	**TS1'$_R$**
	catalyst	0.0	5.2
$\Delta\Delta E_d^{\ddagger}$	styrene	0.0	3.9
	NMs_2^- ···HFIP	0.0	5.3
$\Delta\Delta E_i^{\ddagger}$		0.0	-6.9

[a] Relative energies with respect to the **TS1'$_S$** fragments

Table S8 Computed Relative Gibbs Free Energies (in kcal/mol) of Chiral Complexes and Transition States Involved in Styrene Diamination at Different Level of Theories[a]. Energy Refinements are Done at the **L2** and **L3** Levels of Theory[a] using the Geometries Obtained at the **L1** (SDD$_{(diethylether)}$/M06-2X/6-31G**,SDD(I)) Level of Theory

Stationary Points	L1	L2	L3
**C1$_{si}^+$**	0.0	0.0	0.0
**C1$_{re}^+$**	1.8	4.7	4.4
**C1'$_{si}$**	0.0	0.0	0.0
**C1'$_{re}$**	3.4	6.7	6.0
**TS1'$_{si}$**	0.0	0.0	0.0
**TS1'$_{re}$**	6.5	13.8	18.1

[a] L2=SDD$_{(diethylether)}$/M06-2X/6-311+G**,SDD(I); L3=SDD$_{(diethylether)}$/B3LYP-D3/6-311+G**,SDD(I)

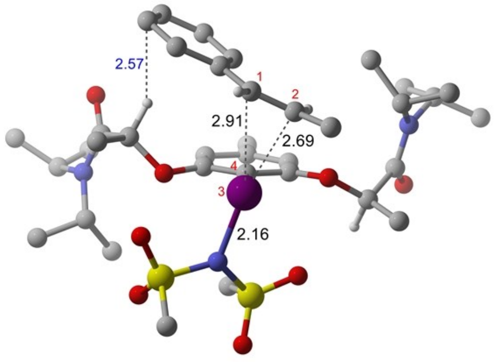

C1$_{\textit{si-re}}^{+}$ (0.0)

$\theta_{(C1\text{-}C2\text{-}I3\text{-}C4)}$ = 96.6

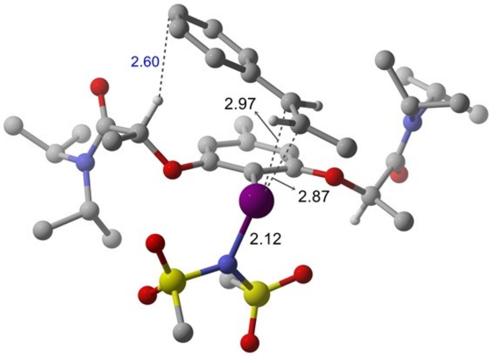

C1$_{\textit{re-si}}^{+}$ (1.9)

$\theta_{(C1\text{-}C2\text{-}I3\text{-}C4)}$ = 9.1

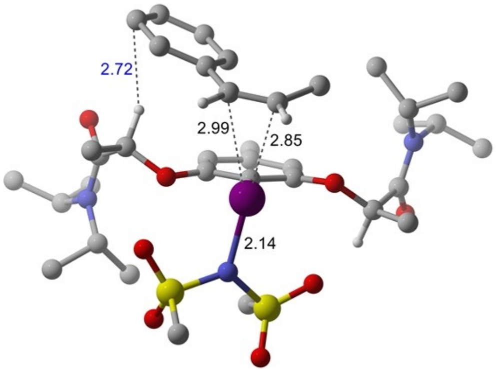

C1$_{\textit{si-si}}^{+}$ (4.8)

$\theta_{(C1\text{-}C2\text{-}I3\text{-}C4)}$ = 80.8

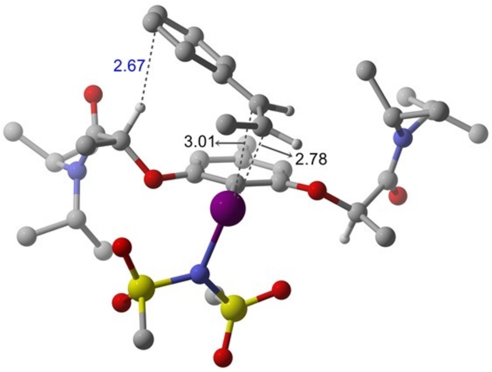

C1$_{\textit{re-re}}^{+}$ (4.9)

$\theta_{(C1\text{-}C2\text{-}I3\text{-}C4)}$ = 11.5

Fig. S12 Optimized geometries of diastereomeric cationic catalyst-substrate complexes of propenyl benzene **S2**. The *trans*-propenyl benzene and the corresponding catalyst-substrate complexes are lower as compared to that for the *cis*-isomer. All distances are in Å, dihedral angles are in degree (º) and relative energies given in parenthesis are in kcal/mol.

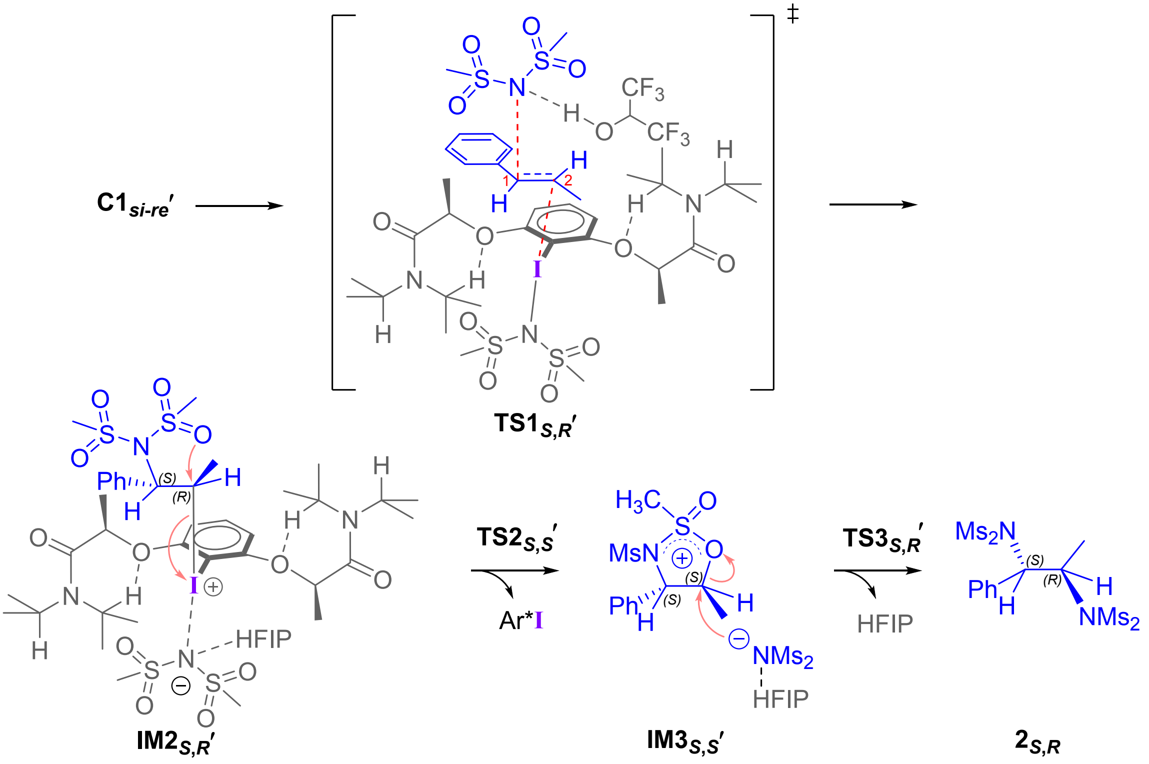

Scheme S3 Formation of 1*S*,2*R*-diaminated product (**2*S,R***) from **C1*si-re*'**. It can be noticed that the nucleophilic addition (**TS1'**) can form stereogenic centers at both C_1 and C_2. Stereogenic center at C_2 is inverted during the intramolecular nucleophilic addition of sulfonyl oxygen (**TS2'**) to form the cyclic oxazolidine oxide intermediate (**IM2**). During the nucleophilic ring opening by imidate (**TS3'**), the stereogenic center at C_2 inverts again, thus product stereochemistry is decided in the nucleophilic addition step. Hence, nucleophilic addition (**TS1*S,R*'**) and consecutive double inversion in the most preferred complex **C1*si-re***$^+$ would lead to a final 1*S*, 2*R*-diaminated product, which is in agreement with experimental observation.

Cartesian Coordinates of the Important Stationary Points for Hypercoordinate Iodine Catalyzed Diamination of Styrene Obtained at the $SDD_{(diethylether)}$/M06-2X/6-31G**,SDD(I) Level of Theory

----

3'...styrene

Electronic energy = -3015.3523395
Zero-point correction= 0.434977 (Hartree/Particle)
Thermal correction to Energy= 0.471736
Thermal correction to Enthalpy= 0.472680
Thermal correction to Gibbs Free Energy= 0.364660
Sum of electronic and zero-point Energies= -3014.917363
Sum of electronic and thermal Energies= -3014.880604
Sum of electronic and thermal Enthalpies= -3014.879660
Sum of electronic and thermal Free Energies= -3014.987679

....

Cartesian Coordinates

....

16	-1.959005	2.406183	1.565656
8	-0.893192	2.169575	2.529554
7	-1.681173	1.290949	0.340336
16	-2.831138	1.069112	-0.864127
8	-3.479047	2.346234	-1.137019
8	-2.128045	0.385762	-1.942495
8	-3.350211	2.306203	1.986298
53	0.179508	0.100684	0.328565
7	2.175770	-0.892219	0.110929
16	3.295625	-0.856799	1.357894
6	1.093801	1.803848	-0.563150
6	2.109124	2.438487	0.137771
6	2.711569	3.543784	-0.462681
6	2.289647	3.980263	-1.716945
6	1.261801	3.319524	-2.387795
6	0.645738	2.208362	-1.812605
16	2.636005	-1.489269	-1.386876
8	3.475063	-2.663431	-1.180827
8	1.394523	-1.616841	-2.140288
8	4.597005	-0.463947	0.829503
8	2.674075	-0.041938	2.396243
1	3.508816	4.062300	0.059444
1	2.423168	2.087516	1.116387
1	-0.152428	1.677300	-2.322810
1	0.936016	3.660114	-3.365090
1	2.764712	4.841503	-2.175280
6	-4.004881	-0.038832	-0.135891
1	-3.474150	-0.950723	0.145563
1	-4.433109	0.461124	0.734386
1	-4.767848	-0.249156	-0.887927
6	3.402497	-2.532526	1.930691
1	3.768628	-3.150735	1.111254
1	4.103184	-2.532577	2.768588
1	2.413529	-2.852913	2.259850
6	3.618238	-0.210851	-2.130928

1	2.994553	0.675759	-2.260039
1	4.469958	-0.010805	-1.480924
1	3.945078	-0.593727	-3.100156
6	-1.692103	3.985838	0.803028
1	-2.427927	4.111865	0.009325
1	-1.819994	4.739362	1.583042
1	-0.673446	4.007065	0.410533
6	-0.623479	-2.672230	2.357517
1	0.292808	-2.573771	2.931704
1	-1.551434	-2.461627	2.884402
6	-0.613292	-3.022393	1.067516
1	0.340394	-3.186736	0.561645
6	-1.821039	-3.164833	0.226911
6	-1.750849	-2.848341	-1.136000
6	-3.043909	-3.592629	0.763628
6	-2.884073	-2.939802	-1.940498
1	-0.806344	-2.513285	-1.559892
6	-4.172023	-3.691686	-0.044755
1	-3.100295	-3.871290	1.812245
6	-4.095126	-3.364615	-1.398812
1	-2.818209	-2.674663	-2.990914
1	-5.110530	-4.035130	0.379766
1	-4.976263	-3.442934	-2.028430

PhI

Electronic energy= -242.9155275
Zero-point correction= 0.090919(Hartree/Particle)
Thermal correction to Energy= 0.096749
Thermal correction to Enthalpy= 0.097693
Thermal correction to Gibbs Free Energy= 0.059223
Sum of electronic and zero-point Energies= -242.824609
Sum of electronic and thermal Energies= -242.818779
Sum of electronic and thermal Enthalpies= -242.817834
Sum of electronic and thermal Free Energies= -242.856305

....

Cartesian Coordinates

....

6	3.348277	-0.000006	0.000002
6	2.650590	-1.205391	-0.000003
6	1.256194	-1.214305	0.000001
6	0.577577	0.000022	-0.000007
6	1.256204	1.214314	-0.000006
6	2.650627	1.205369	0.000004
1	4.433522	-0.000041	0.000003
1	3.187867	-2.148714	0.000003
1	0.712634	-2.152901	0.000001
1	0.712701	2.152943	-0.000008
1	3.187881	2.148705	0.000011
53	-1.559838	-0.000000	0.000001

3'

Electronic energy -2705.8231007
Zero-point correction= 0.298718 (Hartree/Particle)
Thermal correction to Energy= 0.327205

Thermal correction to Enthalpy= 0.328149
Thermal correction to Gibbs Free Energy= 0.237447
Sum of electronic and zero-point Energies= -2705.524382
Sum of electronic and thermal Energies= -2705.495895
Sum of electronic and thermal Enthalpies= -2705.494951
Sum of electronic and thermal Free Energies= -2705.585653

....

Cartesian Coordinates

....

16	2.990372	-0.258095	-1.502161
8	2.008610	-0.573329	-2.529812
7	2.218396	-0.592710	-0.046251
16	3.113411	-0.600064	1.374460
8	4.113569	0.458761	1.320801
8	2.120474	-0.587865	2.442714
8	4.282878	-0.931622	-1.502025
53	0.000697	-0.762245	0.023905
7	-2.214933	-0.568688	0.083714
16	-3.132532	-0.739369	-1.310926
6	-0.000563	1.365060	-0.056647
6	-0.543257	1.966913	-1.182301
6	-0.541446	3.361189	-1.223941
6	-0.008085	4.096016	-0.166333
6	0.527385	3.450594	0.946944
6	0.536053	2.057183	1.017979
16	-2.965924	-0.104560	1.514662
8	-4.226907	-0.825826	1.629219
8	-1.944685	-0.261655	2.539801
8	-4.172051	0.282125	-1.328988
8	-2.162523	-0.786807	-2.398470
1	-0.955336	3.866163	-2.090475
1	-0.949302	1.375744	-1.998202
1	0.943674	1.537749	1.880431
1	0.936587	4.025497	1.771085
1	-0.010752	5.180383	-0.210133
6	3.936956	-2.171383	1.376298
1	3.177505	-2.954074	1.353731
1	4.583622	-2.217521	0.500100
1	4.518032	-2.224060	2.299123
6	-3.898525	-2.328826	-1.147699
1	-4.529509	-2.308343	-0.259152
1	-4.491777	-2.493098	-2.049475
1	-3.113867	-3.080688	-1.060948
6	-3.309801	1.627003	1.326236
1	-2.362720	2.153264	1.191658
1	-3.968720	1.757365	0.468441
1	-3.798305	1.947303	2.249350
6	3.262593	1.497273	-1.498631
1	3.888385	1.746280	-0.642239
1	3.768985	1.734323	-2.436895
1	2.294273	1.999495	-1.449497

---

styrene

Electronic energy= -309.515492
Zero-point correction= 0.134618 (Hartree/Particle)

Thermal correction to Energy= 0.141338
Thermal correction to Enthalpy= 0.142282
Thermal correction to Gibbs Free Energy= 0.103288
Sum of electronic and zero-point Energies= -309.380874
Sum of electronic and thermal Energies= -309.374154
Sum of electronic and thermal Enthalpies= -309.373210
Sum of electronic and thermal Free Energies= -309.412204

Cartesian Coordinates

6	2.255500	0.265151	0.026284
6	1.777040	-1.041890	0.033475
6	0.405994	-1.281653	0.002114
6	-0.511260	-0.224486	-0.031829
6	-0.015424	1.087057	-0.045765
6	1.352638	1.328092	-0.015142
1	3.323695	0.457203	0.048021
1	2.470328	-1.876977	0.062096
1	0.034883	-2.303415	0.007396
1	-0.703587	1.925924	-0.090651
1	1.718460	2.350310	-0.028660
6	-1.953998	-0.533020	-0.054560
1	-2.192991	-1.588512	-0.179833
6	-2.955680	0.338686	0.072471
1	-2.791901	1.402722	0.218101
1	-3.987742	0.005131	0.041241

2

Electronic energy = -2772.5369481
Zero-point correction= 0.349241(Hartree/Particle)
Thermal correction to Energy= 0.376655
Thermal correction to Enthalpy= 0.377600
Thermal correction to Gibbs Free Energy= 0.290263
Sum of electronic and zero-point Energies= -2772.187707
Sum of electronic and thermal Energies= -2772.160293
Sum of electronic and thermal Enthalpies= -2772.159348
Sum of electronic and thermal Free Energies= -2772.246686

Cartesian Coordinates

7	2.021679	-0.183330	-0.178586
16	3.262298	0.865367	-0.678636
16	2.447689	-1.636325	0.614620
8	4.237311	0.867185	0.398223
8	2.603352	2.094696	-1.082309
8	3.699747	-2.125879	0.062687
8	1.251999	-2.463624	0.529451
6	0.703253	-0.129309	-0.841267
6	-0.404048	0.092901	0.202324
1	0.739251	0.670680	-1.578240
1	0.513472	-1.067111	-1.368034
7	-1.668331	-0.549962	-0.240594
16	-2.373643	-1.782838	0.704088
16	-2.505455	0.046092	-1.588556
8	-3.813558	-1.590484	0.651460

8	-1.688991	-1.747859	1.986983
8	-3.162115	-1.087997	-2.216379
8	-1.520673	0.816909	-2.332028
6	-3.721896	1.149761	-0.923891
1	-4.279281	1.547615	-1.775080
1	-3.201350	1.950391	-0.394908
1	-4.371672	0.578708	-0.260284
6	-1.927228	-3.262620	-0.158780
1	-2.361372	-4.099407	0.393012
1	-0.837163	-3.322387	-0.165281
1	-2.341771	-3.198563	-1.165346
6	2.696516	-1.123679	2.290535
1	3.512122	-0.400581	2.303507
1	2.953785	-2.020700	2.858291
1	1.767063	-0.685337	2.658033
6	3.960081	0.102099	-2.120398
1	4.715905	0.790308	-2.504951
1	3.164226	-0.034471	-2.855083
1	4.405467	-0.848435	-1.828146
1	-0.131298	-0.494764	1.079904
6	-0.651578	1.516656	0.673928
6	-1.300437	1.673365	1.904950
6	-0.331278	2.648224	-0.075714
6	-1.628072	2.938504	2.378041
1	-1.556620	0.790044	2.486065
6	-0.656234	3.918091	0.402496
1	0.173372	2.556648	-1.029631
6	-1.305964	4.066915	1.623582
1	-2.130288	3.045315	3.334516
1	-0.398392	4.791977	-0.187447
1	-1.558394	5.057378	1.989576

HFIP

Electronic energy = -789.5385463
Zero-point correction= 0.064348(Hartree/Particle)
Thermal correction to Energy= 0.073221
Thermal correction to Enthalpy= 0.074165
Thermal correction to Gibbs Free Energy= 0.029402
Sum of electronic and zero-point Energies= -789.474198
Sum of electronic and thermal Energies= -789.465325
Sum of electronic and thermal Enthalpies= -789.464381
Sum of electronic and thermal Free Energies= -789.509145

....

Cartesian Coordinates

....

1	0.015870	1.927057	0.765678
8	0.003924	1.873379	-0.202446
6	-0.000074	0.530632	-0.567058
1	-0.001377	0.460859	-1.658172
6	-1.264429	-0.147583	-0.049278
6	1.263057	-0.150130	-0.049654
9	2.343052	0.407001	-0.599554
9	1.283194	-1.456308	-0.319716
9	1.360231	0.002431	1.280242
9	-2.344190	0.468904	-0.531131

9	-1.317355	-0.074220	1.290145
9	-1.329065	-1.433636	-0.396874

HNMs$_2$

Electronic energy = -1232.088825
Zero-point correction= 0.113769 (Hartree/Particle)
Thermal correction to Energy= 0.124147
Thermal correction to Enthalpy= 0.125091
Thermal correction to Gibbs Free Energy= 0.078050
Sum of electronic and zero-point Energies= -1231.975056
Sum of electronic and thermal Energies= -1231.964679
Sum of electronic and thermal Enthalpies= -1231.963734
Sum of electronic and thermal Free Energies= -1232.010775

....

Cartesian Coordinates

....

7	-0.001155	0.035901	0.857763
16	1.475001	0.190879	0.060795
16	-1.471101	-0.188355	0.051691
8	-1.231950	-0.989789	-1.137589
8	-2.377006	-0.656747	1.086047
8	1.221783	1.070674	-1.066968
8	2.423394	0.550979	1.098296
6	1.853845	-1.432337	-0.542118
1	1.067528	-1.730205	-1.236118
1	2.819799	-1.363962	-1.047099
1	1.914174	-2.111053	0.309780
6	-1.893427	1.457647	-0.437629
1	-1.985523	2.068298	0.460828
1	-1.102711	1.827496	-1.091602
1	-2.845486	1.396062	-0.968948
1	0.025624	-0.331136	1.809237

C1

Electronic energy = -3015.3365072
Zero-point correction= 0.435639 (Hartree/Particle)
Thermal correction to Energy= 0.471879
Thermal correction to Enthalpy= 0.472823
Thermal correction to Gibbs Free Energy= 0.365975
Sum of electronic and zero-point Energies= -3014.900868
Sum of electronic and thermal Energies= -3014.864628
Sum of electronic and thermal Enthalpies= -3014.863684
Sum of electronic and thermal Free Energies= -3014.970532

....

Cartesian Coordinates

....

53	0.101744	-0.126158	-0.542773
6	2.145269	0.428811	-0.457641
6	2.712700	0.604443	0.800040
6	4.066876	0.919379	0.872466
1	4.529771	1.064585	1.843108
6	4.818390	1.051981	-0.295043
1	5.873951	1.296585	-0.231354

6	4.225242	0.872752	-1.542760
1	4.813525	0.968882	-2.449543
6	2.869992	0.555370	-1.636028
6	-0.300139	2.261181	-1.591094
6	-0.186349	2.815247	-0.344265
1	0.512582	2.285802	-2.313376
1	-1.085366	2.804780	0.271665
16	1.688217	-3.037590	-1.019179
7	0.774476	-2.103458	0.048453
8	1.576884	-2.352932	-2.299093
16	0.430765	-2.677925	1.601565
8	1.265366	-4.423383	-0.886505
8	1.639032	-3.308123	2.118532
8	-0.130578	-1.530262	2.303664
6	-0.816906	-3.913534	1.390017
1	-1.122007	-4.213986	2.395051
1	-1.637967	-3.461008	0.833197
1	-0.373902	-4.748155	0.847560
6	3.362131	-2.902572	-0.443608
1	3.970603	-3.494864	-1.130734
1	3.658825	-1.852884	-0.473713
1	3.409990	-3.304422	0.567993
6	1.015100	3.391457	0.238582
6	1.046559	3.599539	1.626263
6	2.146873	3.711352	-0.530989
6	2.193938	4.090367	2.238370
1	0.165743	3.361752	2.216871
6	3.288148	4.205841	0.082045
1	2.125005	3.582963	-1.608817
6	3.315062	4.390345	1.466744
1	2.213152	4.243501	3.312315
1	4.159577	4.453461	-0.515607
1	4.210775	4.778888	1.941725
1	-1.291956	1.996361	-1.947584
16	-3.343284	-0.889640	0.044599
7	-2.881823	0.615484	-0.319680
8	-2.191895	-1.733262	-0.362374
16	-3.978993	1.814374	-0.203110
8	-4.649454	-1.263141	-0.506131
8	-4.799304	1.699411	1.009266
8	-3.229924	3.060902	-0.397970
6	-5.069075	1.634760	-1.599934
1	-5.774855	2.467083	-1.572830
1	-4.469212	1.669912	-2.510120
1	-5.583618	0.677960	-1.511898
6	-3.444782	-1.012469	1.816113
1	-3.740296	-2.035801	2.058139
1	-2.457118	-0.792705	2.225340
1	-4.191153	-0.297934	2.164546
1	2.117873	0.495304	1.701968
1	2.402611	0.389461	-2.601119

C1'

Electronic energy= -3804.8959538

Zero-point correction= 0.501439 (Hartree/Particle)

Thermal correction to Energy= 0.548326
Thermal correction to Enthalpy= 0.549271
Thermal correction to Gibbs Free Energy= 0.417780
Sum of electronic and zero-point Energies= -3804.394515
Sum of electronic and thermal Energies= -3804.347627
Sum of electronic and thermal Enthalpies= -3804.346683
Sum of electronic and thermal Free Energies= -3804.478174

....

Cartesian Coordinates

....

16	1.926581	-3.703838	-0.405456
8	3.024587	-4.271855	0.362245
7	1.495507	-2.240243	0.354701
16	1.528605	-2.106148	2.051653
8	0.904183	-0.824800	2.328410
8	0.988537	-3.338188	2.599575
8	2.121873	-3.358359	-1.803272
53	0.951045	-0.562462	-0.822501
6	0.585135	1.308107	-2.773424
6	0.290325	2.272856	-1.859942
6	2.698197	0.475054	-0.229949
6	3.885185	0.241709	-0.916465
6	5.027864	0.917303	-0.493241
6	4.961069	1.800029	0.584035
6	3.756246	2.019129	1.249234
6	2.601327	1.351463	0.846709
1	5.968943	0.749399	-1.006625
1	3.923261	-0.450812	-1.751652
1	1.657691	1.502830	1.359120
1	3.707103	2.711092	2.083643
1	5.855930	2.323913	0.905260
1	-0.722885	2.299303	-1.451294
1	1.554305	1.238500	-3.260735
1	-0.204524	0.672179	-3.165966
6	3.252965	-1.996794	2.455547
1	3.302726	-1.853666	3.537626
1	3.740697	-2.924224	2.158373
1	3.672137	-1.133769	1.934508
6	0.491418	-4.724008	-0.226191
1	0.723459	-5.674752	-0.711699
1	0.310098	-4.859106	0.840617
1	-0.338437	-4.215246	-0.719000
7	-2.218503	-0.135805	-1.503862
16	-2.361662	-1.672664	-2.022149
16	-3.370144	0.959197	-1.894871
8	-3.071118	2.128222	-1.057467
8	-4.719134	0.391984	-1.814076
8	-1.129060	-2.328226	-1.528810
8	-2.630969	-1.760321	-3.459087
6	-3.091503	1.415453	-3.591254
1	-3.888240	2.107744	-3.870481
1	-3.131749	0.509832	-4.198498
1	-2.120109	1.904409	-3.673423
6	-3.712128	-2.423781	-1.143807
1	-3.691746	-3.490299	-1.376123
1	-4.638832	-1.961888	-1.481400

1	-3.558348	-2.252217	-0.076991
1	-2.131082	-0.237406	0.257804
8	-2.231615	-0.388557	1.230460
6	-2.668718	0.797518	1.797146
1	-3.358600	1.362623	1.156571
6	-3.406262	0.452082	3.079503
6	-1.489282	1.725653	2.079731
9	-0.674453	1.750624	1.008791
9	-1.891758	2.984524	2.301762
9	-0.756496	1.344291	3.127959
9	-4.560493	-0.160609	2.796790
9	-2.699745	-0.359431	3.867476
9	-3.687873	1.561728	3.780109
6	1.221526	3.275600	-1.346898
6	0.811324	4.091030	-0.281276
6	2.521563	3.425157	-1.859641
6	1.686028	5.019684	0.273417
1	-0.192842	3.981556	0.116618
6	3.391809	4.349561	-1.302528
1	2.851799	2.821568	-2.699349
6	2.977875	5.145730	-0.231598
1	1.359816	5.642705	1.099818
1	4.395252	4.455063	-1.702716
1	3.663021	5.868167	0.201393

TS1'

Electronic energy= -3804.8870914
Zero-point correction= 0.501085(Hartree/Particle)
Thermal correction to Energy= 0.546984
Thermal correction to Enthalpy= 0.547928
Thermal correction to Gibbs Free Energy= 0.418323
Sum of electronic and zero-point Energies= -3804.386007
Sum of electronic and thermal Energies= -3804.340107
Sum of electronic and thermal Enthalpies= -3804.339163
Sum of electronic and thermal Free Energies= -3804.468769

Cartesian Coordinates

16	4.923529	1.348549	-1.241024
8	3.825392	1.933149	-2.007767
7	4.391017	-0.155144	-0.822912
16	5.391441	-1.220216	-0.067356
8	6.248980	-0.544184	0.906913
8	4.513980	-2.290212	0.414016
8	6.250340	1.269438	-1.851003
53	2.001143	-0.662132	-1.030969
6	-0.279888	-0.795568	-0.958385
6	-0.501198	-1.775502	0.062858
6	1.928631	0.560614	0.707090
6	1.305296	1.796740	0.614333
6	1.276666	2.590906	1.761527
6	1.868600	2.141808	2.939720
6	2.489311	0.893614	2.991206
6	2.526596	0.077597	1.862465
1	0.790986	3.560110	1.719735

1	0.852476	2.141932	-0.310022
1	3.018510	-0.890872	1.877423
1	2.947860	0.546259	3.911340
1	1.847584	2.768624	3.825362
1	-0.453856	-2.818677	-0.251007
1	-0.566839	0.229613	-0.741504
1	-0.544112	-1.131662	-1.958296
6	-0.562460	-1.516340	1.453880
6	-0.886331	-0.234123	1.954850
6	-0.248227	-2.567571	2.349309
6	-0.860526	-0.005987	3.318844
1	-1.205743	0.545040	1.272664
6	-0.198486	-2.320224	3.708688
1	-0.022179	-3.553812	1.952655
6	-0.504181	-1.040440	4.187772
1	-1.128508	0.970193	3.708498
1	0.065652	-3.112313	4.400355
1	-0.479154	-0.853706	5.257295
6	6.429888	-1.879537	-1.348586
1	7.097149	-2.610821	-0.888556
1	5.792549	-2.353093	-2.096168
1	6.991363	-1.051249	-1.782695
6	5.077282	2.265581	0.275435
1	5.422578	3.265477	0.004478
1	4.096206	2.317209	0.751284
1	5.802400	1.759567	0.912500
7	-3.154983	-1.535051	-0.414958
16	-3.423814	-2.538988	-1.684111
16	-4.258196	-1.509851	0.795627
8	-5.634453	-1.559321	0.294642
8	-3.879609	-0.352417	1.617298
8	-4.005622	-3.813040	-1.252730
8	-2.159038	-2.599807	-2.426367
6	-3.980166	-2.967658	1.773156
1	-4.690578	-2.941386	2.601804
1	-2.955809	-2.936772	2.148120
1	-4.147979	-3.838795	1.139879
6	-4.612757	-1.703102	-2.708847
1	-4.803735	-2.337186	-3.576849
1	-4.187005	-0.747296	-3.015904
1	-5.519397	-1.557916	-2.119937
1	-2.804828	0.226700	-0.589466
8	-2.469796	1.156909	-0.673875
6	-3.381115	2.063137	-0.146347
1	-4.018745	1.628051	0.631990
6	-2.574494	3.183267	0.490059
6	-4.281460	2.575092	-1.262194
9	-4.856667	1.531647	-1.875114
9	-5.253699	3.366058	-0.797175
9	-3.597336	3.263896	-2.182836
9	-1.947653	2.730727	1.589276
9	-1.630492	3.654766	-0.331799
9	-3.352408	4.205270	0.857792

TS1

Electronic energy= -3015.3169129
Zero-point correction= 0.435141(Hartree/Particle)
Thermal correction to Energy= 0.470907
Thermal correction to Enthalpy= 0.471851
Thermal correction to Gibbs Free Energy= 0.363185
Sum of electronic and zero-point Energies= -3014.881772
Sum of electronic and thermal Energies= -3014.846006
Sum of electronic and thermal Enthalpies= -3014.845062
Sum of electronic and thermal Free Energies= -3014.953728

....

Cartesian Coordinates

....

53	0.569659	-0.492956	-0.593686
6	1.741533	1.276036	-0.482200
6	2.110308	1.724448	0.779442
6	2.888555	2.877729	0.850481
1	3.195118	3.253255	1.821188
6	3.274704	3.538520	-0.315878
1	3.884056	4.434217	-0.251399
6	2.893149	3.053503	-1.564655
1	3.207306	3.561732	-2.470526
6	2.114065	1.899312	-1.663358
6	-1.235978	0.768232	-1.241168
6	-1.576483	1.392182	-0.002276
1	-0.884202	1.386411	-2.063336
1	-2.062132	0.744159	0.722770
16	3.910657	-1.539729	-1.063941
7	2.698118	-1.457310	0.050504
8	3.268933	-1.212708	-2.336914
16	2.963859	-1.877201	1.621652
8	4.638281	-2.801616	-0.942019
8	4.307317	-1.482648	2.046051
8	1.819726	-1.342006	2.360707
6	2.875811	-3.649597	1.648258
1	3.032050	-3.972531	2.679318
1	1.888799	-3.949672	1.294221
1	3.659728	-4.031998	0.993347
6	5.034524	-0.217512	-0.669834
1	5.820158	-0.235275	-1.428411
1	4.486183	0.726163	-0.707614
1	5.444096	-0.396793	0.323684
6	-1.180652	2.686953	0.424244
6	-1.357151	3.006867	1.789154
6	-0.567852	3.623666	-0.441135
6	-0.904509	4.217743	2.284243
1	-1.841348	2.285606	2.441346
6	-0.124873	4.832864	0.059982
1	-0.451761	3.400190	-1.496360
6	-0.286473	5.123798	1.420068
1	-1.030366	4.461659	3.333128
1	0.345794	5.555396	-0.597769
1	0.068715	6.074143	1.806915
1	-1.924375	-0.032999	-1.511122
16	-3.960481	-2.399793	0.134960
7	-3.582207	-0.842637	-0.127235
8	-2.699818	-3.141520	0.042366

16	-4.743578	0.259634	-0.325651
8	-5.076189	-2.855358	-0.705945
8	-5.819206	0.170149	0.669583
8	-4.032596	1.554290	-0.413505
6	-5.479639	-0.020368	-1.922596
1	-6.221806	0.763316	-2.084597
1	-4.694086	0.034035	-2.677602
1	-5.941754	-1.007517	-1.915843
6	-4.512626	-2.493657	1.825600
1	-4.737704	-3.539433	2.042711
1	-3.711500	-2.133149	2.471919
1	-5.402194	-1.871549	1.927749
1	1.821834	1.179570	1.673635
1	1.828743	1.497810	-2.630025

IM1

Electronic energy = -3015.3573298
Zero-point correction= 0.437986 (Hartree/Particle)
Thermal correction to Energy= 0.473553
Thermal correction to Enthalpy= 0.474498
Thermal correction to Gibbs Free Energy= 0.366941
Sum of electronic and zero-point Energies= -3014.919344
Sum of electronic and thermal Energies= -3014.883776
Sum of electronic and thermal Enthalpies= -3014.882832
Sum of electronic and thermal Free Energies= -3014.990389

....

Cartesian Coordinates

....

53	0.224332	0.372715	-0.444540
6	1.091206	2.293337	-0.140218
6	1.571467	2.586586	1.130301
6	2.142147	3.839670	1.341578
1	2.528204	4.090521	2.324294
6	2.227132	4.757742	0.295059
1	2.677527	5.730353	0.465512
6	1.749771	4.429546	-0.971194
1	1.830810	5.139548	-1.787895
6	1.174183	3.179530	-1.204133
6	-1.775746	1.278864	-0.559685
6	-2.893061	0.497221	0.108625
1	-1.636937	2.228430	-0.041059
1	-2.572225	0.078550	1.068701
16	3.947442	0.208074	-1.078188
7	2.805180	-0.189606	0.007121
8	3.221160	0.601437	-2.288327
16	3.197217	-0.872399	1.422692
8	4.978712	-0.828749	-1.196462
8	4.379392	-0.257627	2.039522
8	1.955297	-0.861514	2.214574
6	3.591195	-2.568763	1.067785
1	3.788112	-3.069759	2.017845
1	2.737837	-3.013645	0.551847
1	4.474277	-2.578593	0.427300
6	4.743486	1.671102	-0.445491
1	5.523110	1.951438	-1.156600

1	3.995812	2.462633	-0.365978
1	5.169502	1.434655	0.530236
6	-4.082379	1.404019	0.318896
6	-4.322793	1.934027	1.585764
6	-4.898893	1.765273	-0.755192
6	-5.375484	2.824642	1.780025
1	-3.689785	1.646218	2.421100
6	-5.954243	2.649311	-0.555986
1	-4.717811	1.345084	-1.740575
6	-6.191701	3.182514	0.710080
1	-5.560048	3.232825	2.768534
1	-6.591036	2.923829	-1.390861
1	-7.013675	3.874775	0.862075
1	-1.989079	1.456766	-1.614739
16	-0.511138	-3.403625	-0.056904
7	-1.381062	-2.048541	-0.483563
8	0.777925	-3.243484	-0.713593
16	-2.937817	-2.069564	-0.331337
8	-1.315096	-4.595496	-0.328975
8	-3.497674	-2.413837	0.966108
8	-3.322502	-0.587920	-0.774920
6	-3.744213	-2.962946	-1.622935
1	-4.817803	-2.791504	-1.530016
1	-3.353167	-2.612313	-2.578256
1	-3.491673	-4.012666	-1.458211
6	-0.279179	-3.253097	1.694218
1	0.289062	-4.130237	2.013215
1	0.288576	-2.339622	1.895869
1	-1.260378	-3.240687	2.172432
1	1.526875	1.846672	1.924281
1	0.819177	2.906154	-2.191980

TS2

Electronic energy= -3015.3187507
Zero-point correction= 0.435313(Hartree/Particle)
Thermal correction to Energy= 0.471012
Thermal correction to Enthalpy= 0.471956
Thermal correction to Gibbs Free Energy= 0.364512
Sum of electronic and zero-point Energies= -3014.883438
Sum of electronic and thermal Energies= -3014.847739
Sum of electronic and thermal Enthalpies= -3014.846795
Sum of electronic and thermal Free Energies= -3014.954238

....

Cartesian Coordinates

....

16	-4.416836	-1.253927	0.884469
8	-3.487874	-2.246613	1.422466
7	-3.640695	-0.611890	-0.415798
16	-4.393136	0.468940	-1.394976
8	-5.226527	1.399788	-0.631467
8	-3.327566	1.031997	-2.229674
8	-5.769351	-1.679569	0.522121
53	-1.193832	-0.992406	-0.666676
6	1.087474	-1.136880	-0.595400
6	1.449299	0.076513	-1.271642

7	3.814793	-0.883022	-0.247547
16	4.302680	-1.337651	1.216202
6	-1.157216	0.473426	0.874486
6	-0.767624	0.078022	2.145618
6	-0.771084	1.047908	3.149187
6	-1.162514	2.353300	2.861921
6	-1.557258	2.708668	1.571921
6	-1.561126	1.761487	0.550503
16	4.823645	-0.305753	-1.363926
8	6.018937	-1.138845	-1.547998
8	3.980044	-0.060691	-2.544873
8	5.312626	-0.444531	1.802206
8	3.064336	-1.534391	1.992140
1	-0.473126	0.770736	4.154899
1	-0.475393	-0.944619	2.360593
1	-1.888015	2.012679	-0.454340
1	-1.866719	3.725509	1.353475
1	-1.166211	3.099099	3.650552
1	1.430347	0.059816	-2.359631
1	1.346697	-1.231806	0.457975
1	1.270775	-2.041311	-1.172772
6	1.684704	1.324286	-0.655456
6	1.901563	1.447245	0.740732
6	1.685457	2.476565	-1.476956
6	2.070165	2.702525	1.292297
1	1.984903	0.556818	1.357776
6	1.842547	3.729209	-0.910803
1	1.549159	2.360789	-2.548667
6	2.028576	3.836916	0.470550
1	2.248189	2.809658	2.356661
1	1.830662	4.618270	-1.530957
1	2.161892	4.819160	0.914286
6	5.381803	1.284537	-0.785974
1	6.026595	1.708564	-1.558153
1	4.508712	1.921358	-0.626251
1	5.926441	1.138431	0.146830
6	5.069574	-2.930289	1.016137
1	5.393087	-3.279158	1.998351
1	4.337234	-3.614028	0.585589
1	5.920262	-2.806076	0.344033
6	-5.463733	-0.495988	-2.432146
1	-5.953965	0.188397	-3.127289
1	-4.855536	-1.224036	-2.969997
1	-6.191595	-0.994032	-1.790613
6	-4.562769	0.055174	2.079899
1	-5.061407	-0.369411	2.953659
1	-3.560660	0.399314	2.342620
1	-5.153575	0.859155	1.641678

IM2'

Electronic energy= -3804.9477769
Zero-point correction= 0.505446 (Hartree/Particle)
Thermal correction to Energy= 0.551033
Thermal correction to Enthalpy= 0.551977
Thermal correction to Gibbs Free Energy= 0.423233

Sum of electronic and zero-point Energies= -3804.442331
Sum of electronic and thermal Energies= -3804.396744
Sum of electronic and thermal Enthalpies= -3804.395800
Sum of electronic and thermal Free Energies= -3804.524544

....

Cartesian Coordinates

....

7	-2.978876	-1.362438	0.406393
16	-3.121745	-2.043218	1.982117
16	-4.328824	-1.534809	-0.629459
8	-3.761683	-3.334031	1.793642
8	-1.790545	-1.960264	2.553350
8	-5.535607	-1.509127	0.178390
8	-4.166329	-0.549292	-1.688383
6	-1.907888	-0.328678	0.241467
6	-1.467533	-0.234146	-1.218690
1	-1.062756	-0.752223	0.793004
1	-2.158887	0.274266	-1.883941
1	-1.132064	-1.188286	-1.630954
16	2.071355	-2.443943	-2.189806
7	1.555622	-1.581841	-0.886074
8	1.467129	-3.779217	-2.218971
8	1.852979	-1.564019	-3.338037
16	1.400855	-2.315873	0.570380
53	0.320718	1.009302	-1.367415
8	2.591193	-3.092973	0.923657
8	1.020790	-1.221661	1.476836
6	-0.768007	2.839883	-1.511473
6	-1.592474	3.063586	-2.607635
6	-0.614367	3.760006	-0.483634
6	-2.310643	4.256451	-2.655637
1	-1.680798	2.331442	-3.404081
6	-1.329258	4.953897	-0.559517
1	0.031068	3.550045	0.362282
6	-2.177773	5.197097	-1.636111
1	-2.966913	4.450350	-3.497765
1	-1.227346	5.685760	0.235274
1	-2.737586	6.125525	-1.683386
6	-2.250941	0.993863	0.893860
6	-3.331033	1.778817	0.479103
6	-1.456404	1.410831	1.963904
6	-3.615592	2.967805	1.143582
1	-3.947304	1.462398	-0.358695
6	-1.746351	2.602192	2.628369
1	-0.618136	0.790559	2.275584
6	-2.827965	3.377966	2.220488
1	-4.454219	3.576929	0.820880
1	-1.130190	2.917076	3.464729
1	-3.058795	4.303672	2.738835
6	-4.204452	-0.968391	2.889870
1	-3.733066	0.012562	2.968898
1	-5.164480	-0.922883	2.376829
1	-4.308159	-1.423281	3.877998
6	-4.098747	-3.153329	-1.307704
1	-4.093213	-3.870443	-0.486734
1	-4.941515	-3.330060	-1.979862

1	-3.157955	-3.163403	-1.859500
6	3.825169	-2.639964	-1.967853
1	4.206902	-3.166194	-2.844788
1	4.276499	-1.650461	-1.884934
1	3.991573	-3.217593	-1.057929
6	0.034974	-3.450671	0.455556
1	-0.147740	-3.829055	1.462463
1	-0.846191	-2.919918	0.089743
1	0.318756	-4.247014	-0.231514
1	2.772132	-0.316067	-0.581303
8	3.400780	0.433717	-0.434098
6	3.740990	0.468397	0.914760
1	3.109528	-0.174270	1.540665
6	3.553900	1.894524	1.409417
6	5.181350	-0.000409	1.070651
9	5.315990	-1.217390	0.533024
9	5.544383	-0.075010	2.356135
9	6.040392	0.816896	0.450014
9	2.250039	2.215077	1.393875
9	4.191729	2.780408	0.639911
9	3.993906	2.045525	2.663657

IM2

Electronic energy= -3015.3832917
Zero-point correction= 0.438339 (Hartree/Particle)
Thermal correction to Energy= 0.473876
Thermal correction to Enthalpy= 0.474820
Thermal correction to Gibbs Free Energy= 0.366995
Sum of electronic and zero-point Energies= -3014.944953
Sum of electronic and thermal Energies= -3014.909415
Sum of electronic and thermal Enthalpies= -3014.908471
Sum of electronic and thermal Free Energies= -3015.016297

....

Cartesian Coordinates

....

7	-3.563994	-0.647604	-0.305078
16	-4.522955	-1.421277	0.888368
16	-4.380659	0.345382	-1.430509
8	-5.719341	-1.879878	0.203540
8	-3.636802	-2.377592	1.531064
8	-5.474553	1.010799	-0.743950
8	-3.358665	1.144756	-2.088548
6	-2.096509	-0.548835	-0.013166
6	-1.273859	-0.551066	-1.298944
1	-1.892847	-1.499596	0.484415
1	-1.263572	0.395693	-1.832088
1	-1.561893	-1.365119	-1.967150
16	3.853436	-1.102942	1.430169
7	3.454130	-0.908685	-0.132415
8	4.674252	0.000744	1.945660
8	2.587599	-1.374524	2.124812
16	4.545477	-0.352962	-1.205200
53	0.825608	-0.933369	-0.838682
8	5.844593	-1.019712	-1.054553
8	3.881437	-0.426417	-2.509492

6	1.212929	1.143109	-0.508656
6	1.086490	2.008470	-1.583886
6	1.584664	1.538927	0.765987
6	1.331284	3.363044	-1.353358
1	0.816322	1.653027	-2.572976
6	1.833818	2.895333	0.965842
1	1.684347	0.819192	1.572569
6	1.704251	3.801654	-0.085266
1	1.240646	4.066286	-2.174808
1	2.127409	3.237465	1.952954
1	1.900155	4.855582	0.084018
6	-1.749564	0.564633	0.957205
6	-1.873361	1.916120	0.616456
6	-1.288222	0.216154	2.228164
6	-1.545190	2.898774	1.544814
1	-2.218478	2.200884	-0.373195
6	-0.959516	1.203291	3.157107
1	-1.185054	-0.833964	2.492269
6	-1.090979	2.545641	2.815933
1	-1.636736	3.945928	1.272813
1	-0.599797	0.916874	4.140385
1	-0.834884	3.317824	3.535083
6	-4.962196	-0.166958	2.065496
1	-4.047055	0.233147	2.505116
1	-5.536645	0.603796	1.552926
1	-5.566006	-0.670249	2.824874
6	-5.033915	-0.810203	-2.600585
1	-5.731365	-1.469228	-2.085187
1	-5.542130	-0.215252	-3.362910
1	-4.202892	-1.365200	-3.036826
6	4.842501	-2.579005	1.516333
1	5.079623	-2.756698	2.566982
1	4.261521	-3.406220	1.107532
1	5.746861	-2.413812	0.929861
6	4.797250	1.374904	-0.845834
1	5.542490	1.742834	-1.553962
1	3.849934	1.898303	-0.986729
1	5.149692	1.470565	0.181296

TS3'

Electronic energy= -3804.9325963
Zero-point correction= 0.503575 (Hartree/Particle)
Thermal correction to Energy= 0.548714
Thermal correction to Enthalpy= 0.549658
Thermal correction to Gibbs Free Energy= 0.421823
Sum of electronic and zero-point Energies= -3804.429021
Sum of electronic and thermal Energies= -3804.383883
Sum of electronic and thermal Enthalpies= -3804.382938
Sum of electronic and thermal Free Energies= -3804.510773

....
Cartesian Coordinates
....

6	-4.485150	-0.507976	-2.992884
6	-3.113699	-0.671604	-2.797765
6	-2.670722	-1.730125	-2.009959

6	-3.558759	-2.631599	-1.428469
6	-4.923863	-2.458887	-1.641126
6	-5.386631	-1.396641	-2.415278
1	-4.839798	0.322294	-3.594773
1	-2.408413	0.020853	-3.246461
1	-3.197411	-3.448540	-0.812815
1	-5.625026	-3.154185	-1.190677
1	-6.452532	-1.261639	-2.568015
53	-0.594659	-1.902267	-1.578843
7	-1.800487	1.981129	0.545308
16	-2.562527	3.167806	-0.430382
16	-0.850289	2.408862	1.849479
8	-3.114037	4.130886	0.505414
8	-3.425147	2.398607	-1.308317
8	-0.162713	3.654231	1.582850
8	-0.082337	1.147636	2.023656
6	-1.888075	0.532261	0.284410
6	-0.483308	-0.008879	0.410950
1	-2.195672	0.475592	-0.766851
1	0.295963	0.482993	-0.163687
1	-0.222799	-0.854329	1.031305
6	-2.940317	-0.173898	1.126336
6	-2.754323	-1.480963	1.581424
6	-4.157337	0.467676	1.374435
6	-3.767142	-2.129767	2.285834
1	-1.831744	-2.018105	1.384150
6	-5.167398	-0.183649	2.074582
1	-4.322408	1.482790	1.024817
6	-4.974636	-1.484951	2.533213
1	-3.606285	-3.143384	2.639121
1	-6.106485	0.327821	2.259726
1	-5.762596	-1.992745	3.080147
6	-1.252775	3.902899	-1.362522
1	-0.723213	3.115505	-1.898943
1	-0.581387	4.411035	-0.670189
1	-1.731437	4.609312	-2.045030
6	-1.933347	2.603338	3.234883
1	-1.308669	2.853195	4.095430
1	-2.462505	1.661782	3.389151
1	-2.617057	3.420097	2.995269
16	2.603706	2.232911	-0.892436
7	3.247814	0.751683	-1.032085
8	3.243389	3.204961	-1.782628
8	1.147513	2.040940	-1.050953
16	4.868230	0.558015	-1.072110
8	5.565600	1.474809	-0.165230
8	5.080700	-0.881222	-0.890287
6	5.351860	0.978436	-2.730024
1	6.428690	0.819525	-2.813215
1	4.811149	0.328819	-3.419015
1	5.095247	2.025002	-2.898594
6	2.868060	2.768534	0.782475
1	2.411669	3.754336	0.884367
1	2.382070	2.053658	1.447252
1	3.943490	2.802498	0.960502
1	2.518078	-0.592410	-0.519882

8	1.995232	-1.390935	-0.175098
6	2.735518	-2.099307	0.757867
1	3.762316	-2.309375	0.432517
6	2.009556	-3.418121	0.963972
6	2.824958	-1.314388	2.062569
9	3.437694	-0.144429	1.847188
9	3.513735	-1.974554	2.999222
9	1.611317	-1.040771	2.566523
9	1.961301	-4.106905	-0.179590
9	0.739356	-3.217148	1.362453
9	2.603940	-4.182534	1.883412

IM3'

Electronic energy= -3562.0434043
Zero-point correction= 0.414084 (Hartree/Particle)
Thermal correction to Energy= 0.451706
Thermal correction to Enthalpy= 0.452650
Thermal correction to Gibbs Free Energy= 0.341865
Sum of electronic and zero-point Energies= -3561.629321
Sum of electronic and thermal Energies= -3561.591698
Sum of electronic and thermal Enthalpies= -3561.590754
Sum of electronic and thermal Free Energies= -3561.701540

....

Cartesian Coordinates

....

7	2.603109	-0.470411	0.185028
16	3.336957	-1.623656	-0.868460
16	2.967557	-0.479958	1.781200
8	4.611174	-1.870718	-0.212213
8	3.259527	-1.027175	-2.180864
8	2.777992	-1.740877	2.447931
8	2.018589	0.706546	2.159450
6	1.516536	0.474985	-0.179131
6	0.895711	0.815010	1.191065
1	0.767736	-0.080556	-0.752557
1	0.137197	0.098035	1.490915
1	0.555517	1.844028	1.261950
6	2.026871	1.689205	-0.931053
6	3.381401	1.988267	-1.060696
6	1.074867	2.551675	-1.482946
6	3.781967	3.150258	-1.719095
1	4.136826	1.309854	-0.675814
6	1.477479	3.711060	-2.135671
1	0.021464	2.303946	-1.401057
6	2.832796	4.016312	-2.252029
1	4.839963	3.370781	-1.820154
1	0.728218	4.374152	-2.556958
1	3.146561	4.919861	-2.765197
6	2.325353	-3.066244	-0.775528
1	2.875647	-3.838622	-1.319723
1	1.364021	-2.864480	-1.246312
1	2.194042	-3.332724	0.273779
6	4.569640	0.185505	2.104723
1	5.293529	-0.547260	1.745656
1	4.632952	0.305579	3.189509

1	4.654979	1.142145	1.589019
16	-1.112329	-2.685859	0.243690
7	-1.953286	-1.534551	-0.519876
8	-0.821578	-3.831796	-0.628877
8	0.056709	-1.996621	0.820186
16	-3.290231	-1.889350	-1.386072
8	-4.103058	-2.922871	-0.738424
8	-3.913849	-0.594701	-1.668085
6	-2.698088	-2.562292	-2.920743
1	-3.566276	-2.788977	-3.542392
1	-2.064576	-1.815691	-3.400914
1	-2.131930	-3.466722	-2.694019
6	-2.099470	-3.272300	1.600355
1	-1.498566	-4.003315	2.144505
1	-2.343654	-2.421070	2.236050
1	-3.003825	-3.724860	1.193608
1	-1.638618	0.016731	-0.352236
8	-1.430151	0.999504	-0.210489
6	-2.573776	1.637716	0.240109
1	-3.486841	1.287626	-0.260610
6	-2.400563	3.117165	-0.061613
6	-2.743947	1.396149	1.736129
9	-2.773136	0.078674	1.976822
9	-3.873118	1.929136	2.211149
9	-1.723561	1.904004	2.446105
9	-2.334329	3.316699	-1.382161
9	-1.264567	3.598332	0.467671
9	-3.413276	3.844520	0.419192

TS1'*o*

Electronic energy= -3804.8823223
Zero-point correction= 0.502231(Hartree/Particle)
Thermal correction to Energy= 0.547766
Thermal correction to Enthalpy= 0.548710
Thermal correction to Gibbs Free Energy= 0.419306
Sum of electronic and zero-point Energies= -3804.380091
Sum of electronic and thermal Energies= -3804.334556
Sum of electronic and thermal Enthalpies= -3804.333612
Sum of electronic and thermal Free Energies= -3804.463016

....

Cartesian Coordinates

....

53	1.879873	0.238702	-1.121305
6	1.626137	-0.321424	0.920560
6	2.247943	-1.479271	1.360924
6	2.058512	-1.835855	2.695288
1	2.527793	-2.737768	3.074347
6	1.266379	-1.048534	3.529877
1	1.118409	-1.339727	4.564656
6	0.662314	0.110892	3.047029
1	0.046481	0.723809	3.696553
6	0.845213	0.500185	1.719836
6	-0.335594	0.061969	-1.404404
6	-0.618144	-1.294067	-1.852537
1	-0.743800	0.327257	-0.430668

1	-0.541893	-1.464714	-2.924702
16	4.714870	1.658301	0.599583
7	4.274008	0.384896	-0.341673
8	3.662262	2.654374	0.406544
16	5.275535	-0.895671	-0.534345
8	6.103070	2.043007	0.336971
8	5.954202	-1.250142	0.714926
8	4.455965	-1.927628	-1.179273
6	6.514440	-0.371195	-1.695689
1	7.180744	-1.218156	-1.870448
1	6.015834	-0.074047	-2.618879
1	7.052323	0.468709	-1.255347
6	4.623249	1.099717	2.287239
1	4.930400	1.939834	2.913517
1	3.590866	0.820417	2.506586
1	5.298199	0.252631	2.409138
6	-0.635647	-2.459938	-1.038469
6	-0.538131	-3.712925	-1.690899
6	-0.791230	-2.411080	0.367246
6	-0.572597	-4.883447	-0.956945
1	-0.429988	-3.740169	-2.771522
6	-0.854557	-3.592301	1.089626
1	-0.904636	-1.463030	0.879382
6	-0.740737	-4.818288	0.431484
1	-0.483111	-5.844251	-1.451255
1	-1.006883	-3.554199	2.162305
1	-0.788041	-5.738429	1.006075
1	-0.559982	0.806628	-2.164718
16	-4.044328	-1.365891	1.508195
7	-3.272071	-0.846498	0.155338
8	-3.029578	-1.353975	2.559788
16	-3.832183	-1.160195	-1.321312
8	-4.779546	-2.609234	1.262530
8	-5.126798	-0.553787	-1.623735
8	-2.712265	-0.735916	-2.224257
6	-4.003331	-2.920147	-1.528216
1	-4.094594	-3.090526	-2.602991
1	-3.125045	-3.423083	-1.123140
1	-4.897565	-3.241708	-0.998426
6	-5.233921	-0.098005	1.879592
1	-5.771711	-0.409171	2.777359
1	-4.701796	0.837482	2.056008
1	-5.913744	-0.004622	1.031180
1	2.860402	-2.078539	0.693633
1	0.379752	1.401842	1.335391
1	-2.607413	0.658919	0.538270
8	-2.236781	1.562350	0.754570
6	-2.532822	2.404885	-0.308021
1	-2.557822	1.894028	-1.282635
6	-3.908519	3.032297	-0.107148
6	-1.440043	3.461700	-0.369286
9	-4.009553	3.635223	1.080183
9	-4.840318	2.073554	-0.158716
9	-4.188462	3.933151	-1.055180
9	-1.403142	4.205501	0.736270
9	-1.597345	4.274391	-1.416674

9	-0.238965	2.867344	-0.494456

TS1$_t$

Electronic energy= -3015.3046291
Zero-point correction= 0.435584 (Hartree/Particle)
Thermal correction to Energy= 0.471000
Thermal correction to Enthalpy= 0.471944
Thermal correction to Gibbs Free Energy= 0.366953
Sum of electronic and zero-point Energies= -3014.869045
Sum of electronic and thermal Energies= -3014.833629
Sum of electronic and thermal Enthalpies= -3014.832685
Sum of electronic and thermal Free Energies= -3014.937676

....

Cartesian Coordinates

....

53	1.177354	-1.002246	-0.231638
6	1.376366	1.085978	-0.586411
6	1.548247	1.905081	0.520751
6	1.683658	3.271796	0.289020
1	1.809647	3.942431	1.132560
6	1.656849	3.772080	-1.012626
1	1.763192	4.838772	-1.181057
6	1.504836	2.914275	-2.099147
1	1.501440	3.303580	-3.111717
6	1.367063	1.540552	-1.896766
6	-1.304881	-0.658515	0.003077
6	-1.228627	-0.883838	-1.386605
1	-1.205984	-1.897893	-1.766014
16	4.122750	-0.801525	1.662556
7	3.535558	-0.839497	0.116092
8	2.937052	-0.842964	2.516127
16	4.522846	-0.535258	-1.176630
8	5.063554	0.304933	1.826772
8	5.762516	-1.297966	-1.048958
8	3.670742	-0.750165	-2.344941
6	4.936745	1.192385	-1.109080
1	5.524552	1.405733	-2.004347
1	4.014067	1.775125	-1.109266
1	5.516117	1.374923	-0.205004
6	5.019766	-2.321363	1.860110
1	5.402369	-2.339087	2.882544
1	4.332413	-3.150756	1.690505
1	5.833483	-2.331277	1.133908
6	-1.508425	0.662186	0.604286
6	-1.300309	0.813031	1.981729
6	-1.905522	1.763670	-0.164469
6	-1.458088	2.057570	2.580027
1	-1.004877	-0.048076	2.576491
6	-2.070332	3.005318	0.440966
1	-2.127487	1.641594	-1.220826
6	-1.837743	3.156240	1.807398
1	-1.290199	2.170749	3.646137
1	-2.388688	3.854658	-0.154613
1	-1.966260	4.127873	2.274041

1	-1.507753	-1.518995	0.635420
16	-5.106763	-0.855494	1.184038
7	-4.050417	-1.102752	-0.032042
8	-4.593675	-1.613206	2.324849
16	-4.397700	-0.551842	-1.491057
8	-6.490991	-1.107029	0.758736
8	-4.953197	0.805992	-1.529092
8	-3.146581	-0.741979	-2.289651
6	-5.595776	-1.640030	-2.228944
1	-5.773988	-1.300846	-3.250808
1	-5.190697	-2.652241	-2.217843
1	-6.502785	-1.579879	-1.626519
6	-5.000073	0.874077	1.599227
1	-5.701610	1.050815	2.417059
1	-3.979838	1.091925	1.918428
1	-5.268960	1.458707	0.719508
1	-0.996334	-0.077135	-2.072366
1	1.577127	1.499897	1.527122
1	1.279288	0.856218	-2.733416

TS4'

Electronic energy = -3562.0231349
Zero-point correction= 0.413035 (Hartree/Particle)
Thermal correction to Energy= 0.450410
Thermal correction to Enthalpy= 0.451355
Thermal correction to Gibbs Free Energy= 0.341872
Sum of electronic and zero-point Energies= -3561.610100
Sum of electronic and thermal Energies= -3561.572724
Sum of electronic and thermal Enthalpies= -3561.571780
Sum of electronic and thermal Free Energies= -3561.681263

....

Cartesian Coordinates

....

7	-1.670949	-1.511258	0.115976
16	-1.583873	-2.457783	1.538648
16	-1.613551	-2.187203	-1.401668
8	-2.286567	-3.684922	1.204735
8	-2.058714	-1.581300	2.589288
8	-0.634830	-3.249334	-1.459529
8	-1.404858	-0.923303	-2.183793
6	-1.821342	-0.043174	0.118801
6	-0.913560	0.454298	-0.995823
1	-1.396686	0.282085	1.069219
1	0.124878	0.159139	-0.996236
7	-0.132832	2.180826	0.106993
16	0.862615	1.889813	1.354489
16	-0.061431	3.628093	-0.673549
8	2.231698	2.394684	1.108863
8	0.755438	0.446002	1.617847
8	-0.000789	4.732471	0.283335
8	-1.167119	3.586156	-1.633123
1	-1.252093	1.211612	-1.691795
6	-3.273090	0.400400	0.008729
6	-4.312502	-0.450721	0.391493
6	-3.571276	1.701547	-0.402300

6	-5.634661	-0.015248	0.338250
1	-4.104935	-1.461973	0.729096
6	-4.894403	2.132843	-0.452368
1	-2.777371	2.388906	-0.679206
6	-5.929755	1.276535	-0.087222
1	-6.432038	-0.689321	0.634713
1	-5.111239	3.145242	-0.778299
1	-6.960015	1.615473	-0.128908
6	0.141729	-2.780685	1.744017
1	0.648340	-1.817490	1.825588
1	0.482383	-3.357568	0.884072
1	0.235255	-3.357860	2.667206
6	-3.209469	-2.820735	-1.825738
1	-3.443317	-3.607429	-1.105924
1	-3.118798	-3.228952	-2.835243
1	-3.929465	-2.002397	-1.790643
6	0.229094	2.770685	2.756384
1	0.249098	3.835102	2.520291
1	0.871358	2.537238	3.607480
1	-0.791863	2.428833	2.932980
6	1.445149	3.634360	-1.620051
1	2.292574	3.688717	-0.937573
1	1.409939	4.520733	-2.256927
1	1.477903	2.730724	-2.231014
1	2.871599	1.429038	-0.241651
8	3.011453	0.658792	-0.827613
6	3.386546	-0.401432	-0.010298
1	3.041709	-0.290931	1.025717
6	4.906132	-0.513312	0.014238
6	2.739573	-1.659702	-0.564275
9	1.404839	-1.505735	-0.592165
9	3.002326	-2.723996	0.203558
9	3.138207	-1.933230	-1.805348
9	5.426289	0.618223	0.501279
9	5.414008	-0.704038	-1.205703
9	5.312599	-1.524619	0.792432

C1'$_{re}$

Electronic energy= -4960.5934135
Zero-point correction= 1.050249 (Hartree/Particle)
Thermal correction to Energy= 1.127948
Thermal correction to Enthalpy= 1.128893
Thermal correction to Gibbs Free Energy= 0.934112
Sum of electronic and zero-point Energies= -4959.543165
Sum of electronic and thermal Energies= -4959.465465
Sum of electronic and thermal Enthalpies= -4959.464521
Sum of electronic and thermal Free Energies= -4959.659302

....

Cartesian Coordinates

....

16	-1.018105	-0.550067	-3.447205
8	0.093325	0.364453	-3.239772
7	-1.108336	-1.451232	-2.005133
16	-2.075329	-2.857512	-1.915467

8	-3.329911	-2.527801	-2.579712
8	-2.070174	-3.237309	-0.514436
8	-1.014901	-1.489409	-4.555161
53	0.031027	-0.854588	-0.339149
6	1.401266	-0.272741	2.062889
6	0.622479	0.817083	2.252435
6	-1.161033	0.869347	-0.232133
6	-0.622655	2.097445	-0.635842
6	-1.468953	3.207240	-0.693838
6	-2.808249	3.096767	-0.309226
6	-3.321749	1.867484	0.112250
6	-2.502057	0.740202	0.139905
1	-1.091080	4.163297	-1.040310
1	-4.365870	1.784699	0.398519
1	0.887297	1.730313	1.718386
1	2.311249	-0.195620	1.480520
1	1.224451	-1.217490	2.567360
6	-0.563390	0.900669	3.108806
6	-1.395198	2.026114	3.009571
6	-0.882478	-0.098688	4.041671
6	-2.518166	2.152658	3.820727
1	-1.162876	2.792862	2.272249
6	-2.002973	0.029956	4.852402
1	-0.237707	-0.965711	4.152124
6	-2.822947	1.155114	4.744927
1	-3.162507	3.020779	3.724215
1	-2.236565	-0.746001	5.574482
1	-3.702893	1.246706	5.372965
6	-2.526588	0.380258	-3.492359
1	-2.494835	0.964297	-4.415323
1	-2.556671	1.038044	-2.621090
1	-3.358769	-0.323376	-3.502264
6	-1.234518	-4.093482	-2.862996
1	-1.888325	-4.968416	-2.841856
1	-0.279966	-4.294339	-2.378446
1	-1.104020	-3.721421	-3.878790
8	0.701817	2.140788	-0.898126
8	-2.942325	-0.500637	0.449790
6	1.140610	2.958499	-1.999952
6	-3.683978	-0.645858	1.674977
6	2.472110	2.409704	-2.479891
1	3.242144	2.445956	-1.706339
1	2.337368	1.372945	-2.794899
6	-3.528973	-2.084520	2.134116
1	-3.945962	-2.788826	1.410766
1	-4.039641	-2.206199	3.093259
1	-2.468062	-2.313998	2.263951
1	2.809184	2.999634	-3.336545
6	-5.163066	-0.213764	1.648818
8	-5.543457	0.417516	2.634667
6	-7.385674	-0.083115	0.659956
1	-7.833849	-0.469297	-0.258916
1	-3.240336	0.026587	2.412307
6	1.178053	4.475096	-1.722985
8	0.668009	5.187541	-2.587781
6	1.680045	6.437700	-0.371447

1	2.164248	6.593240	0.595858
1	0.400000	2.859011	-2.797894
7	-5.983094	-0.553893	0.626869
7	1.742476	4.976313	-0.599615
6	-5.580754	-1.371809	-0.533192
1	-4.538436	-1.649984	-0.394294
6	2.481078	4.177010	0.394121
1	2.425048	3.137594	0.078138
6	-8.172133	-0.674072	1.828403
1	-7.811201	-0.280921	2.778951
1	-9.231797	-0.423201	1.719687
1	-8.080148	-1.764419	1.839582
6	-7.471559	1.441059	0.599245
1	-6.946253	1.819268	-0.284252
1	-8.519048	1.749217	0.526194
1	-7.038493	1.891187	1.494170
6	-5.666403	-0.556118	-1.821631
1	-5.350871	-1.174463	-2.664877
1	-6.686138	-0.208707	-2.019197
1	-5.008637	0.318099	-1.762693
6	-6.389016	-2.665369	-0.614869
1	-7.445752	-2.484606	-0.837077
1	-5.980445	-3.288506	-1.415683
1	-6.322692	-3.223473	0.323962
6	2.474036	7.225468	-1.411965
1	2.502655	8.281810	-1.127167
1	3.503484	6.859242	-1.468803
1	2.014670	7.137895	-2.396986
6	0.238865	6.928137	-0.235399
1	0.237452	7.979083	0.069145
1	-0.294155	6.840553	-1.183594
1	-0.293860	6.353389	0.529703
6	1.821973	4.267533	1.769473
1	1.909447	5.268129	2.204356
1	0.757797	4.016400	1.699031
1	2.303254	3.566090	2.457862
6	3.955344	4.579258	0.431981
1	4.421701	4.432120	-0.546740
1	4.080959	5.627773	0.720525
1	4.489430	3.969287	1.164604
6	-3.690411	4.315410	-0.314406
1	-4.714835	4.060532	-0.596601
1	-3.725724	4.756007	0.688120
1	-3.312542	5.075049	-1.001981
16	2.156820	-3.826161	-0.788885
7	2.448450	-2.922232	0.531895
8	1.391021	-2.926669	-1.679281
16	3.063961	-3.649494	1.867305
8	1.534693	-5.114987	-0.477017
8	4.054936	-4.673868	1.532561
8	3.480404	-2.544693	2.738384
6	1.685086	-4.465284	2.639100
1	2.038363	-4.884641	3.583025
1	0.897039	-3.730709	2.813454
1	1.338453	-5.250407	1.966225
6	3.724411	-4.123120	-1.568536

1	3.531377	-4.663508	-2.497140
1	4.188762	-3.157448	-1.771922
1	4.331815	-4.715596	-0.883385
1	3.322335	-1.518928	0.102196
8	3.801355	-0.688777	-0.173342
6	4.832766	-0.450669	0.726074
1	4.631937	-0.843307	1.732842
6	6.103727	-1.126622	0.231370
6	4.985797	1.056941	0.837730
9	6.477391	-0.677235	-0.971051
9	5.880286	-2.443477	0.123364
9	7.127206	-0.947157	1.073344
9	5.190531	1.635293	-0.349979
9	5.995868	1.403777	1.640682
9	3.860263	1.585232	1.351783

C1$_{re}^{+}$

Electronic energy= -2939.3545221
Zero-point correction= 0.880931 (Hartree/Particle)
Thermal correction to Energy= 0.936993
Thermal correction to Enthalpy= 0.937937
Thermal correction to Gibbs Free Energy= 0.790747
Sum of electronic and zero-point Energies= -2938.473591
Sum of electronic and thermal Energies= -2938.417529
Sum of electronic and thermal Enthalpies= -2938.416585
Sum of electronic and thermal Free Energies= -2938.563775

....

Cartesian Coordinates

....

16	0.337837	3.354643	-1.209192
8	-1.088919	3.155756	-1.007070
7	1.054035	2.779070	0.228488
16	2.712482	3.035606	0.547858
8	3.444400	2.793115	-0.685512
8	2.983334	2.244850	1.735603
8	0.876951	4.681686	-1.448439
53	-0.092725	1.585598	1.565844
6	-1.488613	0.225675	3.492660
6	-1.545254	-0.875095	2.694846
6	-0.336854	0.138283	0.073256
6	-1.573057	0.023577	-0.572547
6	-1.662089	-0.856526	-1.652286
6	-0.555848	-1.624206	-2.033224
6	0.660831	-1.505105	-1.353743
6	0.788797	-0.603737	-0.299203
1	-2.591827	-0.944964	-2.205117
1	1.517659	-2.092570	-1.669153
1	-2.346682	-0.915900	1.957283
1	-2.309085	0.938233	3.482022
1	-0.745916	0.333911	4.279472
6	-0.633310	-2.011903	2.678120
6	-0.742303	-2.929012	1.619865
6	0.329946	-2.229003	3.677939
6	0.091761	-4.039375	1.558337
1	-1.480259	-2.748995	0.840218

6	1.159459	-3.340459	3.615120
1	0.411731	-1.542864	4.515790
6	1.042390	-4.245939	2.556994
1	0.011397	-4.735999	0.730581
1	1.898459	-3.506525	4.392039
1	1.699847	-5.107451	2.507439
6	0.905064	2.264269	-2.484705
1	0.505547	2.662714	-3.420579
1	0.510739	1.264185	-2.294832
1	1.994902	2.282014	-2.487717
6	2.816840	4.756653	0.957305
1	3.858218	4.938647	1.232899
1	2.155211	4.949862	1.802355
1	2.533232	5.341091	0.083013
8	-2.602112	0.737181	-0.073501
8	1.946420	-0.341361	0.344990
6	-3.588694	1.232612	-1.000196
6	2.672093	-1.470773	0.876163
6	-4.250414	2.437949	-0.358327
1	-4.772364	2.178722	0.566289
1	-3.489939	3.191885	-0.145293
6	3.554011	-0.963259	2.001629
1	4.293810	-0.240517	1.650404
1	4.072052	-1.814924	2.450835
1	2.937259	-0.481346	2.764356
1	-4.976575	2.858157	-1.058804
6	3.459014	-2.316378	-0.142544
8	3.349899	-3.534855	-0.008839
6	4.944138	-2.614994	-2.049412
1	5.499566	-1.932500	-2.697185
1	1.937791	-2.174518	1.273964
6	-4.614772	0.196130	-1.507520
8	-4.791751	0.182530	-2.724944
6	-6.295530	-1.543482	-1.223368
1	-6.707123	-2.071526	-0.359795
1	-3.061015	1.545105	-1.905378
7	4.236046	-1.739206	-1.087749
7	-5.297232	-0.605002	-0.657288
6	4.467570	-0.287169	-1.212889
1	3.887586	0.209457	-0.438201
6	-5.157999	-0.588856	0.809071
1	-4.373891	0.128819	1.047631
6	5.965656	-3.524653	-1.369246
1	5.470247	-4.273592	-0.750813
1	6.564196	-4.032577	-2.131517
1	6.642304	-2.938193	-0.740117
6	3.961611	-3.381186	-2.932907
1	3.307760	-2.685945	-3.470199
1	4.511095	-3.967272	-3.675661
1	3.350967	-4.061937	-2.336570
6	3.956934	0.229719	-2.556513
1	4.116489	1.309253	-2.615168
1	4.480390	-0.232325	-3.399827
1	2.885570	0.025881	-2.661369
6	5.935920	0.062330	-0.980351
1	6.588070	-0.345418	-1.759364

1	6.050103	1.150225	-0.984867
1	6.276467	-0.316015	-0.011767
6	-7.455204	-0.815708	-1.900590
1	-8.228757	-1.540229	-2.172356
1	-7.898586	-0.082681	-1.219872
1	-7.121794	-0.303298	-2.803354
6	-5.646137	-2.589832	-2.127602
1	-6.384497	-3.353559	-2.389413
1	-5.274628	-2.136198	-3.047614
1	-4.815365	-3.083941	-1.613208
6	-4.701426	-1.953173	1.328171
1	-5.460045	-2.726081	1.175976
1	-3.784253	-2.270459	0.819761
1	-4.503941	-1.896932	2.403246
6	-6.446891	-0.120068	1.482623
1	-6.745959	0.862783	1.106918
1	-7.270726	-0.819592	1.311293
1	-6.294849	-0.046677	2.563563
6	-0.681370	-2.606662	-3.164702
1	0.226353	-2.622920	-3.772678
1	-0.828408	-3.616367	-2.766677
1	-1.532589	-2.367978	-3.805068

C1'$_{si}$

Electronic energy= -4960.5981422
Zero-point correction= 1.049727 (Hartree/Particle)
Thermal correction to Energy= 1.127642
Thermal correction to Enthalpy= 1.128586
Thermal correction to Gibbs Free Energy= 0.932875
Sum of electronic and zero-point Energies= -4959.548415
Sum of electronic and thermal Energies= -4959.470500
Sum of electronic and thermal Enthalpies= -4959.469556
Sum of electronic and thermal Free Energies= -4959.665267

Cartesian Coordinates

16	-1.045940	-0.466805	-3.474819
8	0.104070	0.397989	-3.254329
7	-1.164401	-1.395715	-2.068959
16	-2.192903	-2.743490	-2.016903
8	-3.448584	-2.351784	-2.649111
8	-2.190652	-3.172768	-0.628180
8	-1.085000	-1.362327	-4.619985
53	0.010528	-0.878632	-0.333166
6	1.312954	0.230209	1.688714
6	0.500260	-0.308620	2.645428
6	-1.106843	0.887772	-0.239813
6	-0.520945	2.096764	-0.636093
6	-1.319249	3.243981	-0.663549
6	-2.656224	3.187057	-0.262234
6	-3.220879	1.974103	0.142760
6	-2.452810	0.812502	0.137240
1	-0.906003	4.187509	-1.003514
1	-4.264127	1.933656	0.441062
1	0.756993	-1.311540	2.987144

1	1.224475	1.261133	1.354268
1	2.228809	-0.295796	1.444871
6	-0.647956	0.308681	3.290195
6	-1.066712	1.619714	3.000879
6	-1.339967	-0.426625	4.268672
6	-2.147390	2.174096	3.672654
1	-0.546955	2.200551	2.245262
6	-2.420602	0.131752	4.940368
1	-1.016778	-1.438356	4.500552
6	-2.826137	1.432325	4.641386
1	-2.465633	3.185263	3.439638
1	-2.949344	-0.444204	5.692500
1	-3.676358	1.866845	5.156705
6	-2.510017	0.532644	-3.496510
1	-2.451674	1.141769	-4.401638
1	-2.516807	1.166251	-2.607588
1	-3.372442	-0.132125	-3.526600
6	-1.424419	-3.987647	-3.016305
1	-2.077159	-4.861347	-2.953569
1	-0.439553	-4.189755	-2.595995
1	-1.359535	-3.615219	-4.037977
8	0.799198	2.088554	-0.923676
8	-2.945788	-0.415733	0.415840
6	1.253343	2.919700	-2.008457
6	-3.713456	-0.567794	1.622533
6	2.545585	2.326350	-2.540382
1	3.337190	2.302658	-1.788700
1	2.353596	1.307818	-2.882690
6	-3.624437	-2.027894	2.029193
1	-4.068789	-2.683726	1.276982
1	-4.143510	-2.164731	2.981739
1	-2.574746	-2.309724	2.149363
1	2.886437	2.927914	-3.387380
6	-5.175264	-0.078327	1.600039
8	-5.537064	0.536282	2.603758
6	-7.395334	0.137078	0.621578
1	-7.852922	-0.207709	-0.308992
1	-3.254762	0.059985	2.389452
6	1.366508	4.421093	-1.678995
8	0.891755	5.189186	-2.515505
6	1.898370	6.298157	-0.222863
1	2.365046	6.390913	0.760859
1	0.492144	2.881825	-2.792520
7	-6.003734	-0.363186	0.569155
7	1.943705	4.851301	-0.532642
6	-5.621512	-1.150648	-0.618384
1	-4.585288	-1.456406	-0.492689
6	2.665690	3.986449	0.418032
1	2.630105	2.972041	0.025161
6	-8.191673	-0.478162	1.770752
1	-7.821004	-0.125483	2.733414
1	-9.246203	-0.201837	1.674008
1	-8.122306	-1.570005	1.743697
6	-7.450109	1.663931	0.615828
1	-6.908013	2.064550	-0.247474
1	-8.490585	1.995384	0.544544

1	-7.016881	2.071763	1.530581
6	-5.692386	-0.290780	-1.878482
1	-5.398455	-0.890175	-2.742840
1	-6.703158	0.090941	-2.057601
1	-5.010134	0.562535	-1.795087
6	-6.457744	-2.422911	-0.741053
1	-7.510031	-2.212442	-0.958678
1	-6.060572	-3.028886	-1.560448
1	-6.405752	-3.011898	0.179717
6	2.723020	7.132277	-1.201677
1	2.774660	8.166784	-0.848594
1	3.743658	6.744592	-1.273480
1	2.272243	7.120141	-2.194677
6	0.462341	6.803348	-0.085703
1	0.471343	7.834896	0.279043
1	-0.053441	6.779545	-1.047284
1	-0.094172	6.193721	0.634245
6	1.970263	3.970692	1.777872
1	2.050002	4.933698	2.292822
1	0.907535	3.738554	1.651903
1	2.424190	3.212833	2.423409
6	4.134971	4.395554	0.520219
1	4.624638	4.323250	-0.455372
1	4.247303	5.419344	0.890023
1	4.656372	3.735014	1.216912
6	-3.493728	4.436934	-0.247585
1	-4.467161	4.260337	-0.713313
1	-3.678635	4.755090	0.783948
1	-2.996834	5.255108	-0.772845
16	2.023148	-3.897106	-0.685695
7	2.255157	-2.862364	0.556733
8	1.248648	-3.107964	-1.664963
16	2.836133	-3.442680	1.973763
8	1.449411	-5.174226	-0.259225
8	3.951656	-4.370432	1.775852
8	3.083407	-2.240792	2.782772
6	1.504006	-4.342751	2.733903
1	1.838274	-4.632527	3.731997
1	0.629917	-3.691848	2.791282
1	1.288891	-5.217966	2.121031
6	3.618921	-4.207271	-1.398247
1	3.467846	-4.849562	-2.267940
1	4.052186	-3.251341	-1.693718
1	4.231643	-4.701523	-0.643227
1	3.288235	-1.539998	0.044452
8	3.854073	-0.783230	-0.258329
6	4.911493	-0.613715	0.627387
1	4.730275	-1.059034	1.615659
6	6.165212	-1.260973	0.053897
6	5.092448	0.883232	0.828935
9	6.511669	-0.724288	-1.120137
9	5.939675	-2.565676	-0.143870
9	7.208766	-1.144759	0.882975
9	5.245628	1.531915	-0.330587
9	6.148506	1.166218	1.597273
9	4.004980	1.391452	1.431937

$C1_{si}^{+}$

Electronic energy= -2939.3588992
Zero-point correction= 0.882259 (Hartree/Particle)
Thermal correction to Energy= 0.937906
Thermal correction to Enthalpy= 0.938851
Thermal correction to Gibbs Free Energy= 0.793982
Sum of electronic and zero-point Energies= -2938.476640
Sum of electronic and thermal Energies= -2938.420993
Sum of electronic and thermal Enthalpies= -2938.420049
Sum of electronic and thermal Free Energies= -2938.564917

....

Cartesian Coordinates

....

16	0.321910	3.399200	-1.157058
8	-1.106815	3.199852	-0.959423
7	1.033137	2.788105	0.254363
16	2.679154	3.038301	0.577327
8	3.426804	2.812082	-0.651777
8	2.949098	2.231236	1.756560
8	0.854930	4.733453	-1.378875
53	-0.139726	1.556637	1.604886
6	-1.809292	0.035402	2.915386
6	-0.812946	-0.727208	3.475176
6	-0.355456	0.152569	0.077526
6	-1.581382	0.057579	-0.595798
6	-1.667449	-0.828111	-1.671210
6	-0.567357	-1.615218	-2.029249
6	0.639543	-1.511916	-1.331519
6	0.765331	-0.606539	-0.280495
1	-2.588550	-0.907517	-2.239851
1	1.487030	-2.122837	-1.624906
1	-0.365421	-0.357480	4.398098
1	-2.383911	-0.306236	2.059108
1	-2.236361	0.850739	3.493693
6	-0.276687	-1.981276	2.985506
6	-0.769781	-2.622508	1.832381
6	0.755210	-2.595126	3.721325
6	-0.246116	-3.843495	1.434602
1	-1.561521	-2.162139	1.249884
6	1.275199	-3.818458	3.319559
1	1.136460	-2.104732	4.613590
6	0.774712	-4.442617	2.176365
1	-0.628247	-4.330147	0.543217
1	2.070602	-4.285598	3.890276
1	1.185712	-5.394329	1.856128
6	0.874271	2.340442	-2.465902
1	0.448857	2.747682	-3.386350
1	0.498507	1.331757	-2.282772
1	1.963243	2.372033	-2.492123
6	2.798051	4.753102	1.012571
1	3.839172	4.922806	1.296203
1	2.133289	4.941111	1.856343
1	2.523135	5.352492	0.145695
8	-2.605284	0.795947	-0.124992

8	1.914315	-0.362195	0.383699
6	-3.593551	1.259965	-1.064490
6	2.662999	-1.496387	0.864424
6	-4.258780	2.484449	-0.463165
1	-4.785442	2.255286	0.466838
1	-3.498996	3.244337	-0.271215
6	3.558766	-1.002155	1.984883
1	4.276395	-0.256380	1.635581
1	4.101741	-1.853295	2.404454
1	2.948106	-0.547276	2.769038
1	-4.982178	2.880708	-1.180296
6	3.441611	-2.308328	-0.188701
8	3.344115	-3.530846	-0.083714
6	4.888222	-2.548818	-2.131782
1	5.424269	-1.845765	-2.773526
1	1.944823	-2.219985	1.257187
6	-4.614663	0.201031	-1.532024
8	-4.817728	0.160729	-2.744589
6	-6.199170	-1.616065	-1.178032
1	-6.552716	-2.154986	-0.295406
1	-3.069181	1.544989	-1.980904
7	4.196497	-1.702633	-1.132852
7	-5.253836	-0.603272	-0.651718
6	4.410569	-0.245803	-1.231656
1	3.832330	0.229414	-0.441993
6	-5.105051	-0.531662	0.811401
1	-4.384175	0.256330	1.020957
6	5.932387	-3.467874	-1.500857
1	5.458730	-4.237864	-0.891443
1	6.515588	-3.950581	-2.290986
1	6.620238	-2.893124	-0.873073
6	3.891435	-3.300022	-3.012962
1	3.192983	-2.600885	-3.485496
1	4.425498	-3.830667	-3.806769
1	3.326896	-4.028775	-2.427645
6	3.882764	0.291146	-2.560627
1	4.034811	1.372255	-2.599592
1	4.401545	-0.150655	-3.417532
1	2.811999	0.081877	-2.659224
6	5.877752	0.114364	-1.009243
1	6.522892	-0.269326	-1.806277
1	5.979792	1.203278	-0.990732
1	6.235432	-0.282190	-0.054177
6	-7.416872	-0.979396	-1.843999
1	-8.140744	-1.758751	-2.100680
1	-7.902420	-0.273017	-1.164043
1	-7.129644	-0.452525	-2.754521
6	-5.500700	-2.635313	-2.077327
1	-6.191898	-3.451936	-2.305967
1	-5.181467	-2.177956	-3.015187
1	-4.627563	-3.062290	-1.572567
6	-4.528970	-1.836240	1.361491
1	-5.240069	-2.664445	1.283280
1	-3.626061	-2.112623	0.807395
1	-4.274143	-1.722742	2.420259
6	-6.420631	-0.147737	1.486183

1	-6.796000	0.800990	1.091793
1	-7.191292	-0.910689	1.340567
1	-6.264002	-0.037210	2.563394
6	-0.669449	-2.561044	-3.193897
1	-0.227489	-2.105432	-4.086528
1	-0.125263	-3.487291	-2.992854
1	-1.710011	-2.802088	-3.420303

TS1'$_{re}$

Electronic energy= -4960.5757654
Zero-point correction= 1.050238(Hartree/Particle)
Thermal correction to Energy= 1.127114
Thermal correction to Enthalpy= 1.128058
Thermal correction to Gibbs Free Energy= 0.935414
Sum of electronic and zero-point Energies= -4959.525528
Sum of electronic and thermal Energies= -4959.448652
Sum of electronic and thermal Enthalpies= -4959.447708
Sum of electronic and thermal Free Energies= -4959.640352

....

Cartesian Coordinates

....

16	-2.949485	2.705464	-2.379267
8	-1.597935	3.160830	-2.045113
7	-2.818048	1.076197	-2.545383
16	-4.052510	0.180399	-3.156860
8	-5.331497	0.560763	-2.547955
8	-3.621038	-1.211868	-3.050165
8	-3.618549	3.302779	-3.535593
53	-0.705816	0.000997	-1.835274
6	1.259607	-0.888008	-1.161868
6	1.230416	-1.155873	0.250382
7	3.477118	-2.405479	0.102790
16	4.059179	-2.639307	1.622838
6	-1.317810	0.694249	0.055441
6	-0.680729	1.799046	0.622743
6	-1.208484	2.329278	1.804604
6	-2.311671	1.735196	2.418619
6	-2.918701	0.616202	1.840396
6	-2.446675	0.105106	0.633861
16	3.614068	-3.620087	-0.993852
8	2.748745	-3.256746	-2.122155
8	3.374904	-4.925343	-0.370056
8	3.638950	-1.433497	2.344437
8	5.484522	-2.976605	1.606576
1	-0.751046	3.201024	2.259344
1	-3.798570	0.176252	2.299458
1	1.672123	-0.394438	0.898159
1	1.982999	-0.124295	-1.423991
1	1.309040	-1.769978	-1.799504
6	0.518207	-2.194701	0.904884
6	0.276327	-2.045502	2.289699
6	0.083262	-3.358972	0.229558
6	-0.421597	-3.024641	2.980285
1	0.642380	-1.155821	2.795542
6	-0.574788	-4.347726	0.937843

1	0.310869	-3.497295	-0.823976
6	-0.837059	-4.174380	2.304893
1	-0.632300	-2.906232	4.037051
1	-0.889177	-5.256834	0.436700
1	-1.370017	-4.949486	2.846569
6	-3.987075	2.980976	-0.962797
1	-4.073773	4.062053	-0.834561
1	-3.514443	2.528771	-0.089114
1	-4.959168	2.530767	-1.165698
6	-4.147105	0.585060	-4.886950
1	-4.914825	-0.058703	-5.320817
1	-3.176141	0.382710	-5.340564
1	-4.411463	1.637206	-4.985248
6	3.216409	-4.017324	2.378369
1	2.144845	-3.938129	2.197445
1	3.430893	-3.939179	3.446435
1	3.605492	-4.942864	1.962334
6	5.292352	-3.619380	-1.578454
1	5.953942	-3.701741	-0.714980
1	5.473547	-2.696372	-2.124980
1	5.393893	-4.484860	-2.236244
8	0.445326	2.242784	0.024878
8	-3.067751	-0.877829	-0.056008
6	0.647282	3.665470	-0.068778
6	-3.322954	-2.115225	0.622336
6	1.637905	3.891363	-1.196272
1	2.595445	3.401132	-0.998918
1	1.218190	3.494486	-2.123447
6	-3.561445	-3.164619	-0.449197
1	-4.412479	-2.899455	-1.080363
1	-3.746315	-4.131179	0.027846
1	-2.679502	-3.242859	-1.089708
1	1.809864	4.963868	-1.316008
6	-4.443722	-2.138453	1.681898
8	-4.173516	-2.756935	2.711858
6	-6.691077	-1.670443	2.497756
1	-7.564230	-1.167521	2.074604
1	-2.432567	-2.365525	1.204245
6	1.052897	4.391829	1.228674
8	0.500748	5.475705	1.415845
6	2.178262	4.556338	3.381333
1	2.891446	3.925591	3.917274
1	-0.311859	4.116277	-0.332649
7	-5.649222	-1.568646	1.451954
7	1.953638	3.867137	2.091185
6	-6.032210	-0.903942	0.189360
1	-5.162335	-0.901789	-0.463204
6	2.739276	2.644357	1.843800
1	2.505866	2.299609	0.838368
6	-7.097773	-3.116125	2.779708
1	-6.293358	-3.658564	3.276910
1	-7.983542	-3.127132	3.422274
1	-7.347713	-3.632401	1.847666
6	-6.294555	-0.910568	3.762024
1	-6.090602	0.140574	3.531740
1	-7.114211	-0.943802	4.486369

1	-5.408412	-1.355255	4.218554
6	-6.411773	0.556052	0.429935
1	-6.636992	1.024223	-0.531002
1	-7.293328	0.654078	1.072479
1	-5.582128	1.100676	0.893786
6	-7.142194	-1.669041	-0.528356
1	-8.085744	-1.650982	0.027436
1	-7.314313	-1.206165	-1.504054
1	-6.855331	-2.713074	-0.687875
6	2.823762	5.929732	3.207717
1	3.104053	6.329197	4.187464
1	3.730172	5.854410	2.599288
1	2.133590	6.623686	2.727036
6	0.905149	4.603481	4.225378
1	1.131295	5.026235	5.208976
1	0.142452	5.222887	3.749125
1	0.508371	3.593427	4.374914
6	2.345781	1.530566	2.809862
1	2.574304	1.793866	3.848802
1	1.269535	1.332919	2.741148
1	2.899209	0.618583	2.568982
6	4.238598	2.933759	1.888718
1	4.510578	3.695540	1.149830
1	4.564759	3.283910	2.872953
1	4.784467	2.012725	1.672002
1	3.873802	-0.624583	-0.129106
8	3.881891	0.367840	-0.089372
6	4.862254	0.885228	-0.923175
1	4.904800	1.973558	-0.803653
6	4.500513	0.599894	-2.377862
6	6.232586	0.334599	-0.539963
9	5.437198	0.991870	-3.239303
9	3.361396	1.241072	-2.686684
9	4.274810	-0.709559	-2.564811
9	6.248141	-1.001894	-0.622256
9	7.202794	0.810709	-1.325594
9	6.522013	0.666145	0.719710
6	-2.864404	2.298106	3.700182
1	-2.776030	1.566887	4.509712
1	-2.337423	3.207623	3.995740
1	-3.926907	2.535023	3.588575

TS1'*si*

Electronic energy= -4960.5877607
Zero-point correction= 1.051127 (Hartree/Particle)
Thermal correction to Energy= 1.127768
Thermal correction to Enthalpy= 1.128712
Thermal correction to Gibbs Free Energy= 0.936402
Sum of electronic and zero-point Energies= -4959.536634
Sum of electronic and thermal Energies= -4959.459992
Sum of electronic and thermal Enthalpies= -4959.459048
Sum of electronic and thermal Free Energies= -4959.651358

....

Cartesian Coordinates

....

16	-2.804075	2.634900	-2.669460
8	-1.714290	3.143056	-1.834400
7	-2.799059	1.001273	-2.418131
16	-3.714513	0.024936	-3.382927
8	-5.050452	0.597086	-3.569142
8	-3.602248	-1.313301	-2.804544
8	-2.784684	2.957045	-4.097261
53	-0.662643	0.057107	-1.562388
6	1.243635	-0.663350	-0.544872
6	0.947672	-2.015626	-0.170779
7	3.369448	-2.800395	-0.133215
16	4.266636	-2.580057	1.225111
6	-1.368962	0.684563	0.301764
6	-0.857046	1.865763	0.850907
6	-1.373460	2.292986	2.075502
6	-2.298811	1.500652	2.762813
6	-2.742572	0.288827	2.228827
6	-2.300148	-0.118765	0.967425
16	3.393613	-4.273581	-0.865169
8	2.303418	-4.227194	-1.847297
8	3.375976	-5.366761	0.110286
8	4.173692	-1.137945	1.491041
8	5.597530	-3.174964	1.099289
1	-1.049779	3.234056	2.508856
1	-3.426744	-0.329710	2.799361
1	0.988578	-2.755881	-0.969003
1	1.420770	0.065998	0.245222
1	1.944136	-0.594235	-1.372976
6	0.450197	-2.459555	1.085739
6	0.279978	-1.590227	2.186718
6	0.181065	-3.840592	1.231559
6	-0.156955	-2.095441	3.400485
1	0.496038	-0.531878	2.082808
6	-0.246059	-4.337725	2.450925
1	0.339205	-4.502114	0.382993
6	-0.412078	-3.464875	3.531471
1	-0.295636	-1.431034	4.246673
1	-0.445210	-5.396884	2.570278
1	-0.747207	-3.855813	4.487176
6	-4.331546	3.234673	-1.994106
1	-4.326221	4.318147	-2.131323
1	-4.360196	2.980537	-0.934254
1	-5.155558	2.775486	-2.540036
6	-2.921946	-0.007997	-4.976354
1	-3.506659	-0.678996	-5.608674
1	-1.907376	-0.391714	-4.854948
1	-2.912742	1.005106	-5.377684
6	3.446988	-3.426520	2.556048
1	2.478993	-2.959825	2.737331
1	4.088883	-3.313841	3.432312
1	3.344871	-4.476677	2.282961
6	4.930500	-4.334238	-1.756270
1	5.742597	-4.273815	-1.031129
1	4.960901	-3.489245	-2.446213
1	4.956336	-5.278433	-2.303664
8	0.154915	2.463620	0.191892

8	-2.695742	-1.229860	0.320110
6	0.267911	3.894807	0.265241
6	-3.203220	-2.342135	1.061767
6	1.102046	4.354419	-0.917673
1	2.131923	3.994128	-0.865579
1	0.637632	3.995397	-1.837832
6	-3.248131	-3.515590	0.097736
1	-3.936583	-3.323566	-0.729287
1	-3.571182	-4.411644	0.633646
1	-2.251617	-3.690862	-0.316253
1	1.118156	5.447352	-0.929158
6	-4.557489	-2.155053	1.778405
8	-4.611218	-2.608339	2.923352
6	-6.870461	-1.416265	1.912546
1	-7.547509	-0.907349	1.221933
1	-2.504196	-2.548628	1.879807
6	0.802288	4.453173	1.598678
8	0.195839	5.421985	2.055640
6	2.276702	4.430949	3.536843
1	3.118096	3.806834	3.846484
1	-0.739811	4.309176	0.181094
7	-5.605902	-1.573298	1.159895
7	1.888214	3.916686	2.203656
6	-5.580944	-1.054480	-0.221948
1	-4.599587	-1.264424	-0.639564
6	2.670145	2.784510	1.674241
1	2.305613	2.583112	0.667430
6	-7.505202	-2.761129	2.260201
1	-6.890427	-3.307805	2.976137
1	-8.495264	-2.597303	2.696589
1	-7.626773	-3.371271	1.359853
6	-6.698340	-0.508337	3.129260
1	-6.264377	0.452026	2.831577
1	-7.674692	-0.314534	3.583777
1	-6.053084	-0.975265	3.875356
6	-5.759683	0.461954	-0.237572
1	-5.720555	0.811329	-1.272096
1	-6.722996	0.768177	0.184992
1	-4.960073	0.949030	0.330919
6	-6.603500	-1.760090	-1.108786
1	-7.635265	-1.553481	-0.805312
1	-6.475660	-1.404286	-2.135229
1	-6.448019	-2.843383	-1.096293
6	2.775788	5.872777	3.475792
1	3.174151	6.165099	4.452299
1	3.576890	5.971513	2.737198
1	1.964428	6.550594	3.208266
6	1.169983	4.233717	4.572916
1	1.550212	4.494331	5.565374
1	0.308237	4.866094	4.353625
1	0.848251	3.187061	4.597275
6	2.432682	1.535423	2.519961
1	2.824937	1.658250	3.535411
1	1.356867	1.340442	2.595645
1	2.931437	0.663586	2.083644
6	4.153343	3.133120	1.556664

1	4.294677	4.029477	0.945848
1	4.617871	3.297521	2.533214
1	4.688586	2.308953	1.077347
6	-2.783254	1.946090	4.116574
1	-3.677414	1.397653	4.419621
1	-2.009047	1.776071	4.872551
1	-3.009231	3.015507	4.115818
1	3.820596	-1.555468	-1.296638
8	3.992319	-0.822474	-1.947652
6	4.456627	0.280400	-1.239222
1	4.085058	0.321446	-0.206092
6	3.968583	1.526057	-1.960763
6	5.978318	0.233093	-1.159015
9	6.365145	-0.970779	-0.734026
9	6.454257	1.152263	-0.308683
9	6.547225	0.450657	-2.351480
9	2.634257	1.642398	-1.814111
9	4.225465	1.490484	-3.266261
9	4.523433	2.634842	-1.455178
